# BCL2L13 promotes mitophagy through DNM1L-mediated mitochondrial fission in glioblastoma

**DOI:** 10.1038/s41419-023-06112-4

**Published:** 2023-09-02

**Authors:** Jiwei Wang, Anbin Chen, Zhiwei Xue, Junzhi Liu, Ying He, Guowei Liu, Zhimin Zhao, Wenjie Li, Qing Zhang, Anjing Chen, Jian Wang, Xingang Li, Xinyu Wang, Bin Huang

**Affiliations:** 1 Department of Neurosurgery, Qilu Hospital, Cheeloo College of Medicine and Institute of Brain and Brain-Inspired Science, Shandong University, 250012 Jinan, China; 2 Jinan Microecological Biomedicine Shandong Laboratory and Shandong Key Laboratory of Brain Function Remodeling, 250117 Jinan, China; 3 Department of Neurosurgery, Xinhua Hospital Affiliated to Shanghai Jiaotong University School of Medicine, 200092 Shanghai, China; 4 Laboratory of Basic Medical Sciences, Qilu Hospital of Shandong University, Jinan, Shandong China; 5 Department of Biomedicine, University of Bergen, Bergen, Norway

**Keywords:** CNS cancer, Prognostic markers

## Abstract

There is an urgent need for novel diagnostic and therapeutic strategies for patients with Glioblastoma multiforme (GBM). Previous studies have shown that BCL2 like 13 (BCL2L13) is a member of the BCL2 family regulating cell growth and apoptosis in different types of tumors. However, the clinical significance, biological role, and potential mechanism in GBM remain unexplored. In this study, we showed that BCL2L13 expression is significantly upregulated in GBM cell lines and clinical GBM tissue samples. Mechanistically, BCL2L13 targeted DNM1L at the Ser616 site, leading to mitochondrial fission and high mitophagy flux. Functionally, these alterations significantly promoted the proliferation and invasion of GBM cells both in vitro and in vivo. Overall, our findings demonstrated that BCL2L13 plays a significant role in promoting mitophagy via DNM1L-mediated mitochondrial fission in GBM. Therefore, the regulation and biological function of BCL2L13 render it a candidate molecular target for treating GBM.

## Background

Glioblastoma multiforme (GBM) is the most aggressive primary human brain tumor in adults. Despite advances in combination treatments consisting of radiation and chemotherapy following surgical resection, the median patient survival is 14.6 months from initial diagnosis [1, 2]. A better understanding of molecular mechanisms underlying the malignant progression of GBM, therefore, is critical.

BCL2 like 13 (BCL2L13), a BLC2-like protein encoded by the BCL2L13 gene on the human chromosome, was discovered 20 years ago but only recently started catching attention and is likely to become a research hotspot [3]. It is anchored to the mitochondrial outer membrane and can be found in various tissues and cells, including cancer cells. BCL2L13 expression is dramatically increased in proximal gastric cancer compared to distal gastric cancer tissues, which may make it a signature of malignant progression in stomach neoplasms [4]. Moreover, the lack of BCL2L13 is associated with a non-response to preoperative chemoradiotherapy (pCRT) in locally advanced rectal cancer patients, providing a potential strategy to predict pCRT efficacy in rectal cancer patients [5]. BCL2L13 also showed a significant association with tumorigenesis and poor prognosis in leukemia [6–8]. In node-negative breast cancer and lung adenocarcinoma, BCL2L13 is negatively associated with tumor aggressive behaviors [9, 10], indicating that BCL2L13 plays different roles based on the specific cell type. In the glioma research field, Jensen et al. revealed that BCL2L13 is overexpressed in GBM, inhibiting tumor cell apoptosis by preventing MOMP and caspase-3 activation [11]. In this study, we confirmed that BCL2L13 is highly expressed in glioma compared to normal brain tissue.

Moreover, upregulation of BCL2L13 correlated with tumor histological grades in glioma. We further revealed that BCL2L13 supports migration and invasion of GBM cells. However, the biological role and regulatory mechanism of BCL2L13 in GBM remain largely unclear.

Autophagy is an evolutionarily conserved process that maintains control of intracellular components through lysosome-mediated degradation. Mitophagy, a selective type of autophagy, specifically degrades damaged or dysfunctional mitochondria and maintains mitochondrial dynamics and cellular homeostasis [12, 13]. In cellular and tissue repair processes, autophagy/mitophagy principally serves as a protective mechanism against stresses and diverse pathologies, including cancer cells [14]. For GBM, temozolomide treatment, the standard first-line chemotherapy, and radiotherapy induce autophagy, which is considered an escape mechanism of cell survival [15–17]. Our previous work showed that Flavokawain B (FKB), a natural kava chalcone, inhibits the malignant behavior of GBM cells and induces cytoprotective autophagy through the ATF4-DDIT3-TRIB3-AKT-MTOR-RPS6KB1 signaling pathway. The protective FKB-induced autophagy helps GBM evade apoptosis and remain in a senescent state, which effectively promotes cell survival [18]. However, the role of autophagy in cancer is still controversial as it may suppress tumors during cancer development but promote cell survival during cancer progression [19]. The specific role of autophagy, therefore, seems to be highly dependent on cell type and context. Thus, the molecular mechanism and function of autophagy/mitophagy in GBM still need further exploration. In this study, our KEGG analysis results suggested that BCL2L13 is closely related to autophagy/mitophagy in GBM. We further demonstrated that BCL2L13 promotes mitochondrial fission-dependent protective mitophagy in GBM cells, suggesting that BCL2L13 is a potential therapeutic target in GBM.

## Results

### BCL2L13 expression is elevated in GBM

**Fig. 1:**
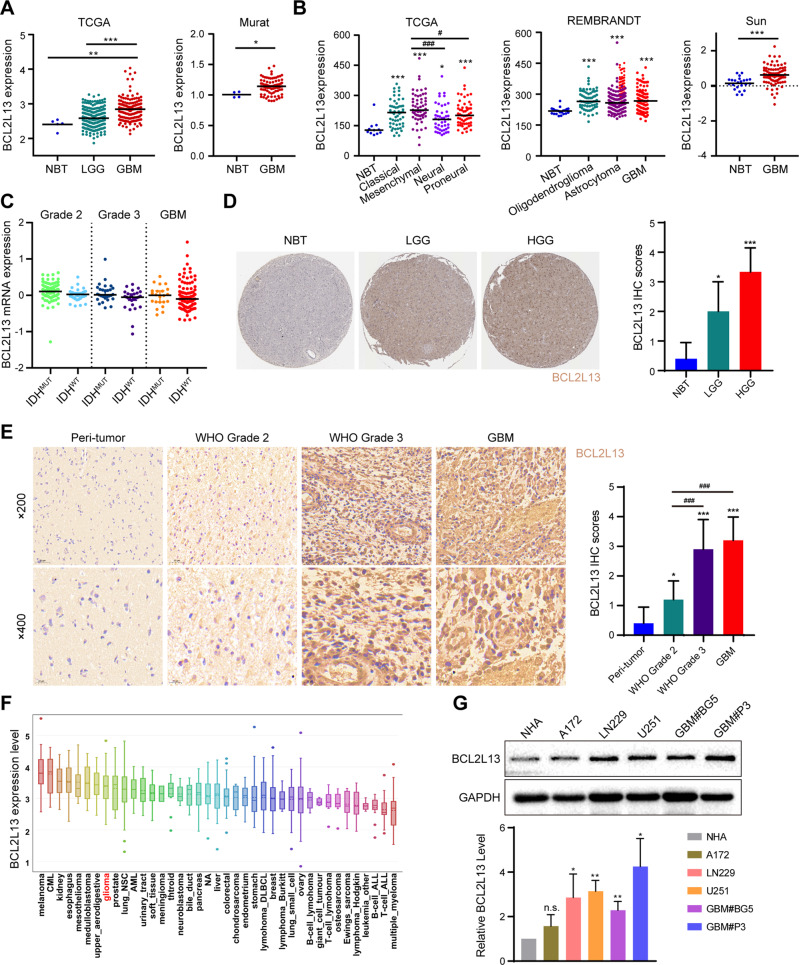
BCL2L13 Expression is elevated in GBM. **A** BCL2L13 mRNA expression as determined using TCGA, Rembrandt, and Oncomine databases. **B** BCL2L13 mRNA expression in different molecular subtypes (classical, mesenchymal, neural, and proneural) from the TCGA GBM microarray dataset. **C** Analysis of BCL2L13 mRNA levels in WHO grade II, grade III, and grade IV gliomas from the TCGA as a function of IDH mutation status. **D** Representative images and statistical results of IHC staining for BCL2L13 from the Protein Atlas database. **E** Representative images of IHC staining for BCL2L13 in different grade gliomas and normal brain tissue. Magnification: ×200, upper; ×400, lower. **F** BCL2L13 mRNA expression across cancer cell lines from different tissues of origin. Glioma cell lines are highlighted in red. **G** BCL2L13 expression in different types of human glioma cell lines. *P < 0.05, **P < 0.01, ***P < 0.001, and ****P < 0.0001 compared to controls. ^#^*P* < 0.05, and ^###^*P* < 0.001 compared between the two treatments indicated.

Microarray data from four publicly available glioblastoma datasets, The Cancer Genome Atlas (TCGA), Rembrandt, Murat Brain, and Sun Brain, were systematically retrieved to begin verifying the level of BCL2L13 in normal brain tissue (NBT) and GBM, and the mRNA level of BCL2L13 was significantly upregulated in GBM compared to NBT (Fig. 1A). Gliomas were molecularly categorized into four subtypes: classical, mesenchymal, proneural, and neural. Classical and mesenchymal subtypes are commonly IDH mutated with poorer survival outcomes than proneural and neural subtypes. In the TCGA database, increased BCL2L13 levels were associated with the mesenchymal subtype compared to proneural or neural subtypes (Fig. 1B). However, the expression of BCL2L13 has no relationship with IDH mutation status in different grades of glioma in the TCGA database (Fig. 1C). Immunohistochemistry (IHC) data from The Human Protein Atlas revealed that increased BCL2L13 levels were associated with increased glioma grade (Fig. 1D). IHC performed on primary glioma sections from our Qilu Hospital further confirmed that BCL2L13 was highly expressed in grade II/III astrocytoma and glioblastoma. In contrast, staining was weak or absent in peri-tumor tissues (Fig. 1E). Next, the expression level of BCL2L13 in cell lines was detected to determine the biological functions of BCL2L13 in GBM. Data derived from the CCLE indicated that glioma cell lines possess a higher expression of BCL2L13 than most cancer cell lines derived from other lineages (Fig. 1F). Western blot was then used to verify BCL2L13 expression levels in different commonly used glioma cell lines. Our results showed that the expression levels of the BCL2L13 protein were increased in several glioma cells, especially in GBM#P3, which is derived from a primary GBM patients [20, 21] (Fig. 1G).

### BCL2L13 knockdown inhibits migration and invasion in GBM cells in vitro

**Fig. 2:**
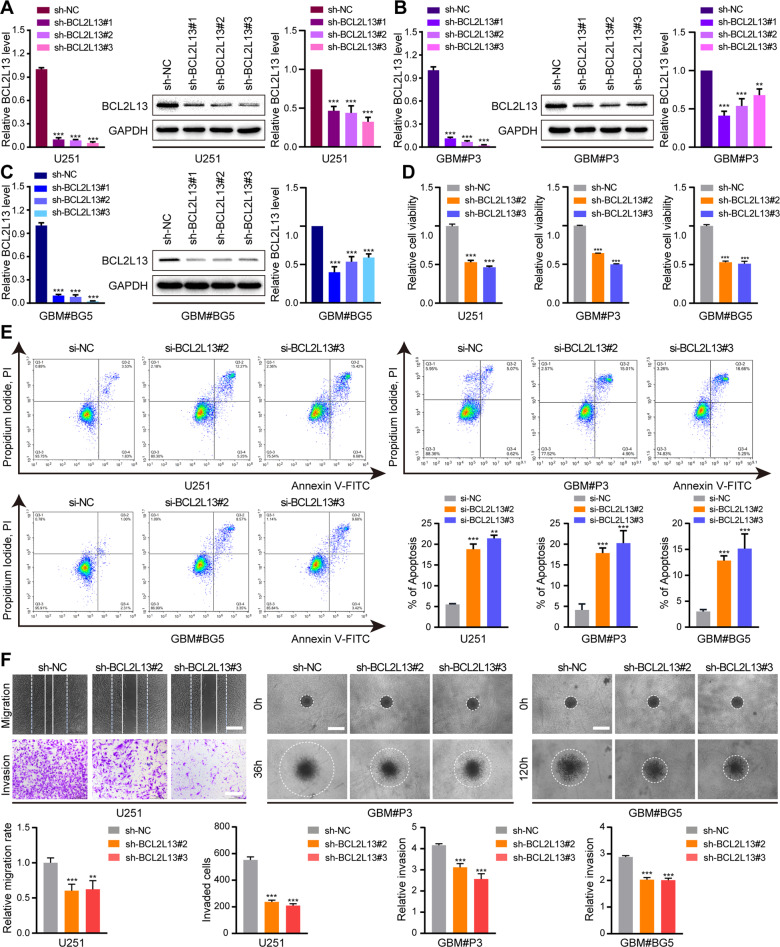
BCL2L13 knockdown inhibits migration and invasion in GBM cells in vitro. **A**–**C** BCL2L13 knockdown efficiency assessed by qRT-PCR and western blotting analysis in U251, GBM#P3, and GBM#BG5 cells transfected with sh-NC or three shRNAs targeting BCL2L13 (sh-BCL2L13#1, #2, and #3). **D** CCK-8 assay for cell viability of U251, GBM#P3 and GBM#BG5 cells transfected with sh-NC or three shRNAs targeting BCL2L13 (sh-BCL2L13#2, and #3) (*n* = 3). **E** Flow cytometry analysis of Annexin V-FITC and propidium iodide (PI) staining for the detection of apoptosis in U251, GBM#P3 and GBM#BG5 cells transfected with siNC, siBCL2L13#2 and siBCL2L13#3 (*n* = 3). **F** Migratory and invasion ability of sh-NC/BCL2L13 U251, GBM#P3, and GBM#BG5 cells were evaluated by wound healing (scale bars: 400 μm) and transwell assay (scale bars: 200 μm). Invasion ability of sh-NC/BCL2L13 GBM#P3, and GBM#BG5 cells were evaluated by 3D invasion (scale bars: 400 μm). Images were taken under bright-field microscopy. All data are expressed as the mean ± SD of values from experiments performed in triplicate.

Quantitative real-time PCR (qRT-PCR) and western blot results showed that BCL2L13 levels in U251, GBM#P3, and GBM#BG5 cells were significantly decreased after infection lentiviruses encoding one of three BCL2L13, especially the case of sh-BCL2L13#2 and #3 (Fig. 2A–C). These two shRNAs were, therefore, selected for the subsequent biological functional assays. We confirmed that U251, GBM#P3 and GBM#BG5 transfected with lentiviruses against BCL2L13 exhibited reduced cell viability (Fig. 2D). Consistent with the CCK-8 experiments, flow cytometry apoptosis assays showed that U251, GBM#P3 and GBM#BG5 transfected with lentiviruses against BCL2L13 increased apoptotic cell death (Fig. 2E). Abnormal migration and invasion are hallmark characteristics of GBM. Wound healing and transwell assays were, therefore, performed to test the role of BCL2L13 in GBM. The wound-healing assay results demonstrated that the migration ability was significantly decreased after BCL2L13 knockdown in U251 (Fig. 2F). Transwell assay and 3D invasion further revealed that knockdown of BCL2L13 led to a significantly lower invasion ability in U251, GBM#P3, and GBM#BG5 cells (Fig. 2F).

### Pathway analysis of BCL2L13 and co‐regulated genes in GBM

**Fig. 3:**
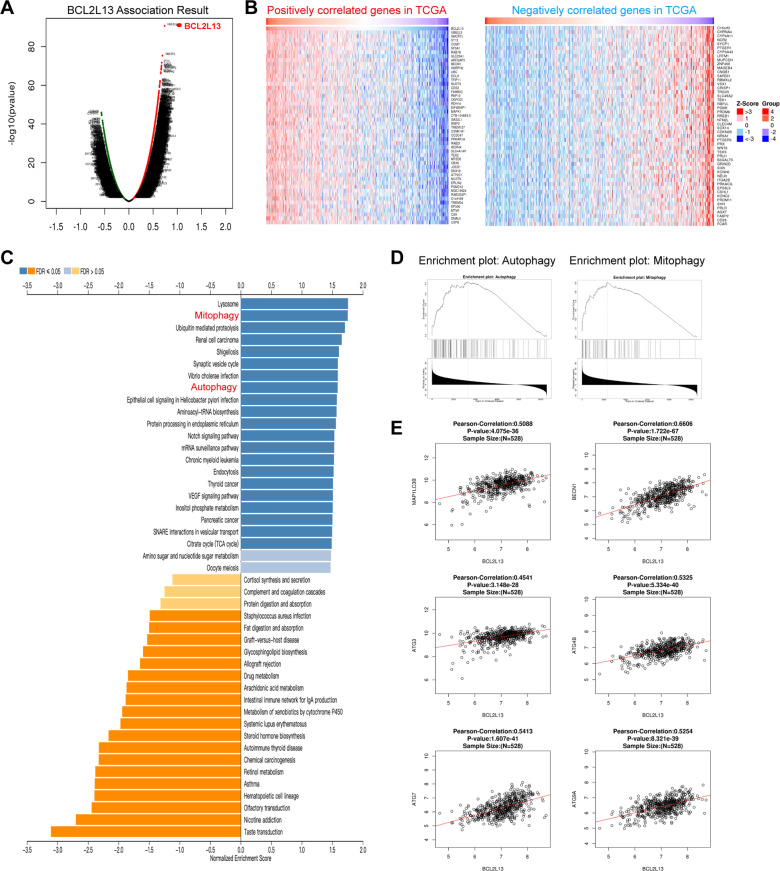
Pathway analysis of BCL2L13 and co‐regulated genes in GBM. **A** Volcano plot of genes correlated with BCL2L13 expression in the TCGA database. **B** Correlation analysis using TCGA data revealing positive and negatively correlated genes with BCL2L13 mRNA expression in human gliomas. **C** KEGG pathway analysis of the positive and negatively correlated genes of BCL2L13 in the TCGA database is illustrated. **D** GSEA highlighting positive association of increased BCL2L13 expression levels with autophagy and mitophagy. **E** Correlation between BCL2L13 and autophagy-related genes in gliomas determined using TCGA dataset.

To further understand the biological processes and mechanism of BCL2L13 in GBM, correlation analysis of BCL2L13 in whole-genome gene profiling was then performed in the TCGA database. Significant genes positively and negatively correlated with BCL2L13 were represented in the volcano plot and heatmaps (Fig. 3A, B). Subsequently, KEGG analysis revealed that BCL2L13-correlated genes were enriched in several important pathways and biological processes, including those related to the lysosome, mitophagy, and autophagy (Fig. 3C). Similar results were observed through the Gene Set Enrichment Analysis (GSEA) in GBM patients (Fig. 3D). We then investigated the relationship between BCL2L13 and the main autophagy-related genes in GBM. Correlation analysis revealed strong linear associations between BCL2L13 and important autophagy-related genes, including MAP1LC3B, BECN1, ATG3, ATG4B, ATG7, ATG9A (Fig. 3E), ULK1, ATG2A, ATG2B, ATG5, ATG12, and BNIP3 (Supplementary Fig. S1) in GBM from TCGA database, highly suggesting that BCL2L13 mediates autophagy in GBM.

### BCL2L13 promotes mitophagy in GBM cells in vitro

**Fig. 4:**
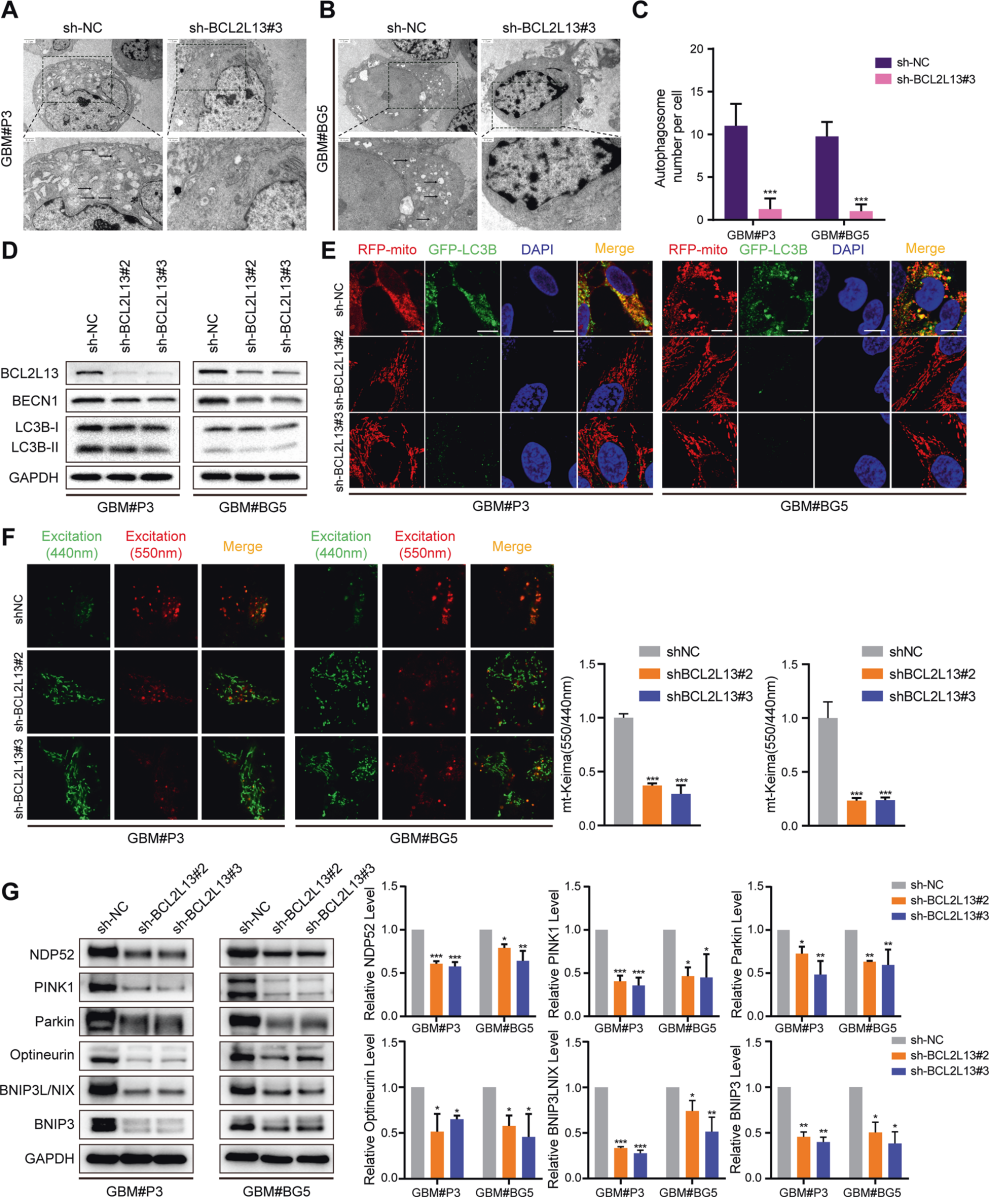
BCL2L13 knockdown inhibits mitophagy in GBM cells in vitro. **A**–**C** TEM images of sh-NC/BCL2L13 GBM#P3 and GBM#BG5 cells. Arrows highlight autophagosomes (arrows). The scale bar in the original image represents 0.8 μm, and the scale bar in the enlarged image represents 0.3 μm. **D** Western blotting analysis to detect protein levels of LC3B, BECN1, and GAPDH (control for loading) in sh-NC/BCL2L13 GBM#P3 and GBM#BG5 cells. Data are representative of three independent experiments. **E** Confocal microscopy images of sh-NC/BCL2L13 GBM#P3 and GBM#BG5 cells after co-expressing RFP-mito and GFP-LC3B (scale bars: 10 μm). **F** Representative confocal images are of GBM#P3 and GBM#BG5 cells expressing mt-Keima transfected with sh-NC or three shRNAs targeting BCL2L13 (sh-BCL2L13#2, and #3). **G** Western blotting analysis to detect protein levels of NDP52, PINK1, Parkin, Optineurin, BNIP3/NIX, BNIP3, and GAPDH (control for loading) in sh-NC/BCL2L13 GBM#P3 and GBM#BG5 cells. All data are expressed as the mean ± SD of values from experiments performed in triplicate.

Transmission electron microscopy (TEM) was performed to detect autophagic vesicles and determine whether BCL2L13 promotes autophagy in GBM cells. TEM analysis demonstrated a decreased production of autophagosomes in GBM#P3 and GBM#BG5 cells knockdown for BCL2L13 (Fig. 4A–C). The western blotting analysis also indicated that the BCL2L13 knockdown inhibited the formation of autophagosomes. BECN1 and MAP1LC3B-II, two markers for autophagy, decreased after BCL2L13 knockdown in both GBM#P3 and GBM#BG5 cells (Fig. 4D and Supplementary Fig. S2A). To confirm whether BCL2L13 affects mitophagy, we used fluorescent-labeled markers for mitochondria (RFP-mito) and autophagosomes (GFP-LC3B) to assess the colocalization of autophagosomes with mitochondria in both GBM#P3 and GBM#BG5 cells. We found a decreased accumulation of MAP1LC3B puncta and colocalization of GFP-LC3B and RFP-mito in BCL2L13 knockdown GBM#P3 and GBM#BG5 cells (Fig. 4E and Supplementary Fig. S2B), indicating that BCL2L13 knockdown influence the process of mitophagy in GBM cells. Furthermore, we explored the effect of BCL2L13 knockdown on mt-keima reporter expression. In BCL2L13 knockdown GBM#P3 and GBM#BG5 cells, the fluorescence of 550 nm excitation light was significantly weakened while that of 440 nm excitation light was enhanced, indicating impaired mitochondrial autophagy (Fig. 4F). Moreover, the expression of mitochondrial markers (NDP52, PINK1, Parkin, Optineurin, BNIP3L/NIX, BNIP3) also decreased in BCL2L13 knockdown GBM#P3 and GBM#BG5 cells (Fig. 4G).

**Fig. 5:**
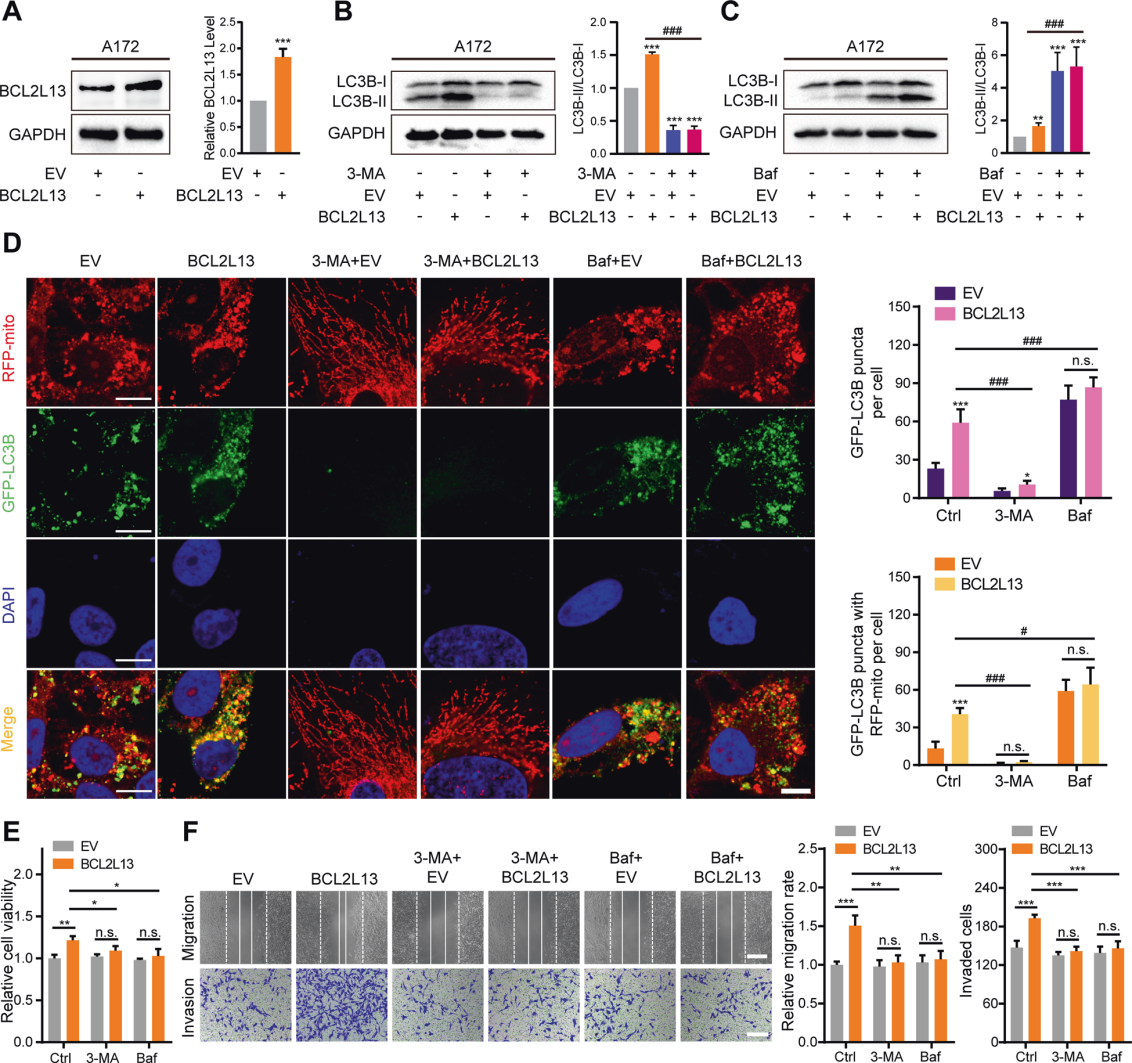
BCL2L13 overexpression promotes mitophagy in GBM cells in vitro. **A** Overexpression of BCL2L13 in A172 cells (A172-*BCL2L13*) was confirmed by western blot. **B**, **C** Western blotting analysis was performed to detect levels of LC3B in A172-EV/BCL2L13 cells after exposure to 3-MA (10 mM) or Baf (100 nM) for 48 h. **D** Confocal microscopy images of autophagy inhibitors (3-MA or Baf) pretreated A172-EV/BCL2L13 cells after co-expressing RFP-mito and GFP-LC3B (scale bars: 10 μm). **E** Cell viability of A172-BCL2L13 cells after autophagy inhibitors (3-MA or Baf) incubation. **F** Representative images and statistic results of migration and invasion ability of A172-EV/BCL2L13 cells after autophagy inhibitors (3-MA or Baf) incubation. All data are expressed as the mean ± SD of values from experiments performed in triplicate. *P < 0.05, **P < 0.01, and ***P < 0.001 compared between the two treatments indicated.

A172, with lower BCL2L13 expression compared with other glioma cells (Fig. 1G), was chosen for ectopic expression experiments. The western blotting analysis showed that expression constructs led to significant increases in BCL2L13 in A172 cells (Fig. 5A). Autophagic flux was used to evaluate BCL2L13 mediated autophagy further. We treated GBM#P3 cells with autophagy inhibitors, 3-MA or bafilomycin A1 (Baf), which block upstream or downstream steps of the process, respectively. The western blotting analysis demonstrated that 3-MA (10 mM) treatment for 48 h led to decreased BCL2L13-induced LC3B-II formation in A172 cells (Fig. 5B). BCL2L13 overexpression induced LC3B puncta, and colocalization of GFP-LC3B and RFP-mito was also reduced (Fig. 5D). In contrast, the incubation of A172 cells with Baf (100 nM) for 48 h still led to increased conversion of LC3B-II (Fig. 5C), accumulation of LC3B puncta, and colocalization of GFP-LC3B and RFP-mito (Fig. 5D).

Finally, to determine whether BCL2L13-induced autophagy protects tumor cells, cell viability, migration, and invasion ability were assessed in the presence of inhibitors of autophagy, 3-MA or Baf, in BCL2L13-overexpressing A172 cells. Cell viability was first assessed using the CCK-8 assay. As shown in Fig. 5E, cell viability was dramatically decreased in A172 cells overexpressing BCL2L13 upon treatment with both 3-MA and Baf (Fig. 5E). Moreover, decreased migration and invasion ability were observed in A172 cells overexpressing BCL2L13 after 3-MA and Baf treatment (Fig. 5F). These results indicate that BCL2L13 induces protective mitophagy in GBM cells.

### BCL2L13 promotes mitochondrial fission in GBM cells through phosphorylation of DNM1L at the Ser616 site

**Fig. 6:**
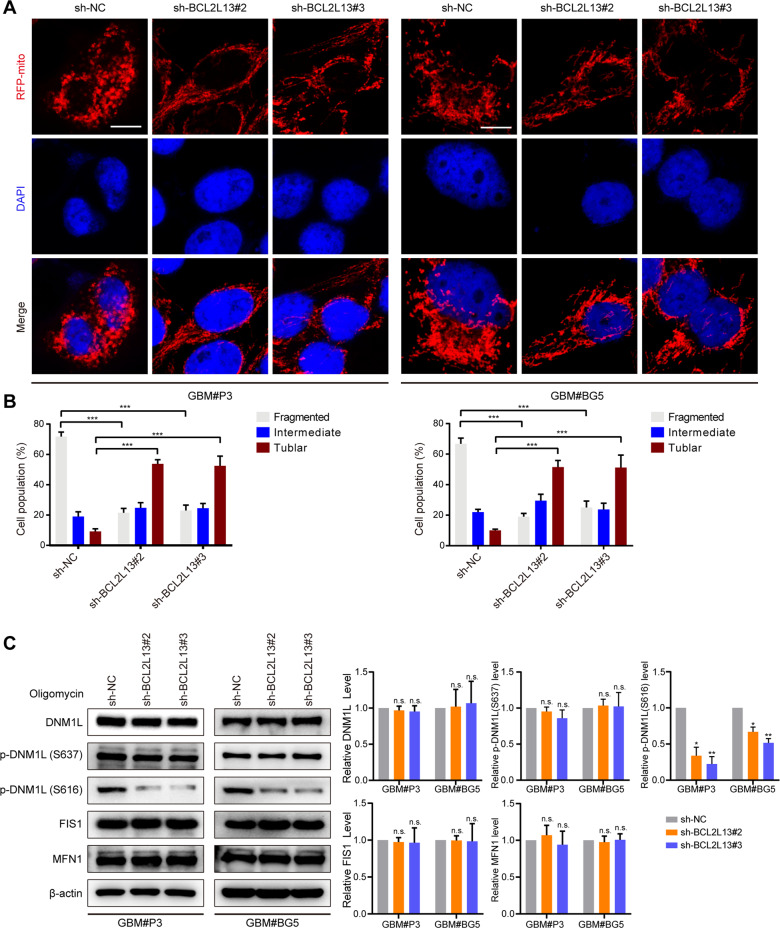
BCL2L13 promotes mitochondrial fission in GBM cells through phosphorylation of DNM1L at Ser616. **A**, **B** Confocal microscopy analysis of mitochondrial morphology was visualized with MitoTracker Red in sh-NC/BCL2L13 GBM#P3 and GBM#BG5 cells. **C** Western blotting analysis for DNM1L, phosphorylated DNM1L at S637 (pS637-DNM1L) and S616 (pS616-DNM1L), FIS1, and MFN1 in sh-NC/BCL2L13 GBM#P3 and GBM#BG5 cells treated with oligomycin. All data are expressed as the mean ± SD of values from experiments performed in triplicate. ***P < 0.001 compared between the two treatments indicated.

Having observed profound changes of mitochondrial morphology in BCL2L13-modified GBM cells, we then quantified the morphological changes of mitochondria in GBM cells knockdown for BCL2L13. Extensive elongation of mitochondria was observed in both GBM#P3 and GBM#BG5 cells with BCL2L13 knockdown (Fig. 6A, B), indicating a change in mitochondrial fission to fusion. We specifically induced mitophagy with oligomycin to monitor the effect of BCL2L13 knockdown on DNM1L phosphorylation in GBM cells. The expression levels of the main mitochondrial dynamic regulatory proteins were then determined by western blotting. As shown in Fig. 6C, no statistical changes in the expression of total DNM1L, DNM1L (S637) phosphorylation, FIS1, and MFN1 were observed in GBM#P3 and GBM#BG5 cells after BCL2L13 knockdown. However, DNM1L (S616) phosphorylation, which has been proved to regulate DNM1L function in the process of mitochondrial fission positively, was significantly decreased in BCL2L13 knockdown GBM cells (Fig. 6C), indicating that BCL2L13 promotes mitochondrial fission in GBM cells by facilitating the phosphorylation of DNM1L (S616).

### BCL2L13 promotes mitochondrial fission-dependent mitophagy in GBM cells

**Fig. 7:**
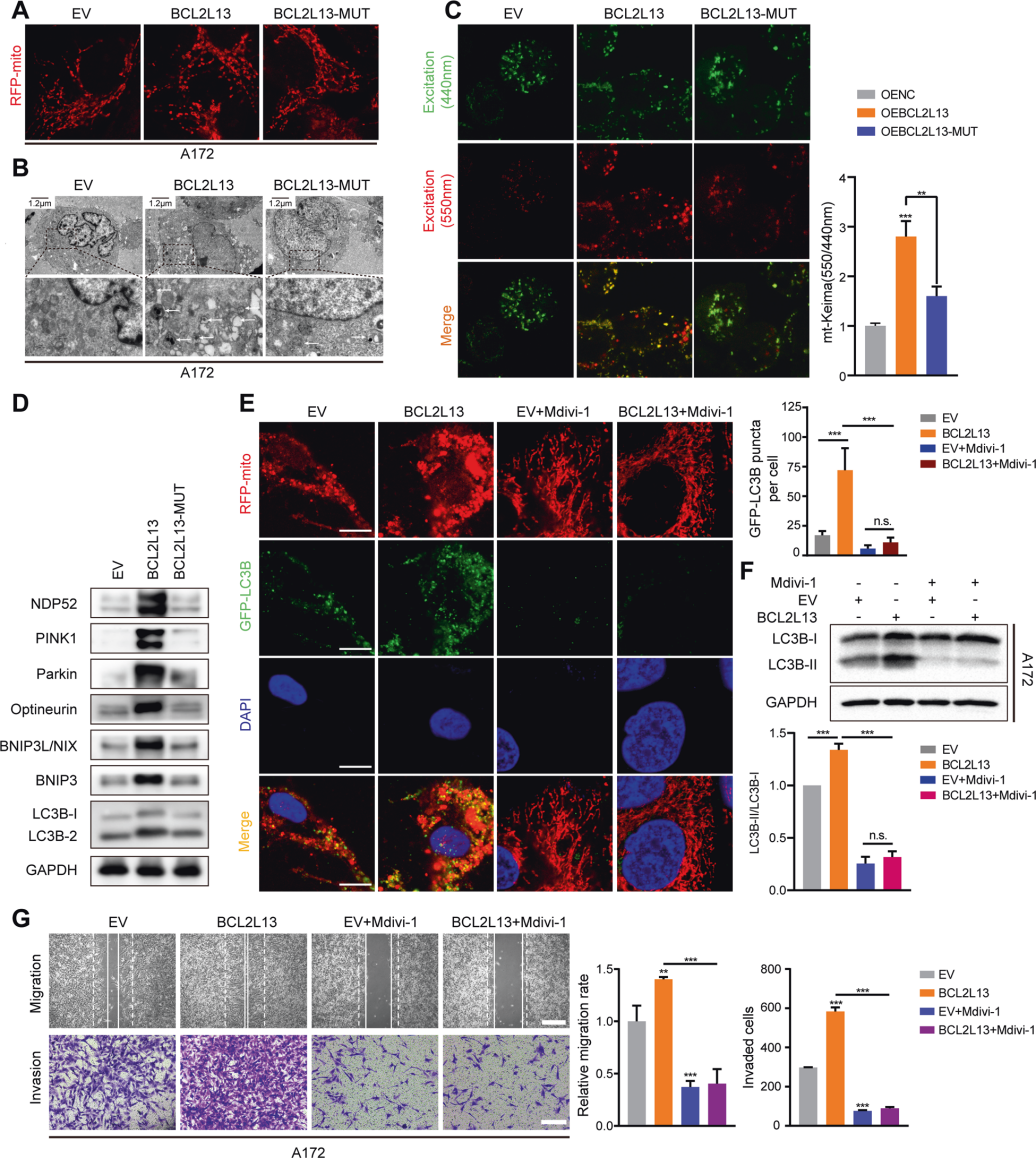
BCL2L13 promotes mitochondrial fission-dependent mitophagy in GBM cells. **A** Confocal microscopy analysis of mitochondrial morphology was visualized with MitoTracker Red in A172-EV/BCL2L13/BCL2L13-MUT cells. **B** TEM images of A172-EV/BCL2L13/BCL2L13-MUT cells. Arrows highlight autophagosomes (arrows). The scale bar in the original image represents 0.8 μm, and the scale bar in the enlarged image represents 0.3 μm. **C** Representative confocal images are of A172 cells expressing mt-Keima transfected with EV/BCL2L13/BCL2L13-MUT. **D** Western blotting analysis to detect protein levels of NDP52, PINK1, Parkin, Optineurin, BNIP3/NIX, BNIP3, LC3B and GAPDH (control for loading) in A172 cells transfected with EV/BCL2L13/BCL2L13-MUT. **E** Confocal microscopy images of GFP-LC3B and RFP-mito expressing A172-EV/BCL2L13 cells after Mdivi-1 (50 µM) treatment for 24 h (scale bars: 10 μm). **F** Western blotting analysis for LC3B in A172-EV/BCL2L13 cells after Mdivi-1 treatment. **G** Representative images and statistic results of migration and invasion ability of A172-EV/BCL2L13 cells treated with mdivi-1. All data are expressed as the mean ± SD of values from experiments performed in triplicate. ***P < 0.001 compared between the two treatments indicated.

Next, we found great fragmentation of mitochondria in A172 cells after BCL2L13 overexpression (Fig. 7A, B and Supplementary Fig. S3A, B). Similarly, the fluorescence of 550 nm excitation light was significantly enhanced while that of 440 nm excitation light was weakened in mt-Keima reporter assay, indicating enhanced mitochondrial autophagy in BCL2L13 overexpression GBM cells (Fig. 7C). Moreover, in BCL2L13 overexpression A172 cells, the expression of mitochondrial markers (NDP52, PINK1, Parkin, Optineurin, BNIP3L/NIX, BNIP3) also increased (Fig. 7D and Supplementary Fig. S3C). However, in BCL2L13-deltaLIR mutant GBM cells, the enhanced mitophagy was abolished (Fig. 7A–D), indicating that LIR motif is necessary for the BCL2L13 enhanced mitophagy. Mdivi-1, a selective mitochondrial fission inhibitor, was used to illustrate further the role of mitochondrial fission in BCL2L13-induced mitophagy. Treatment with Mdivi-1 significantly inhibited mitochondrial fragmentation and decreased the LC3B puncta and colocalization of GFP-LC3B and RFP-mito in BCL2L13 overexpressed A172 cells (Fig. 7E and Supplementary Fig. S3D). Moreover, Mdivi-1 treatment led to decreased BCL2L13-induced LC3B-II formation in A172 cells (Fig. 7F). Next, we validated that Mdivi-1 could inhibit GBM cell migration and invasion (Fig. 7G). These results indicate that BCL2L13 promotes mitochondrial fission-dependent mitophagy in GBM cells.

### BCL2L13 knockdown inhibits GBM growth in an orthotopic mouse model

**Fig. 8:**
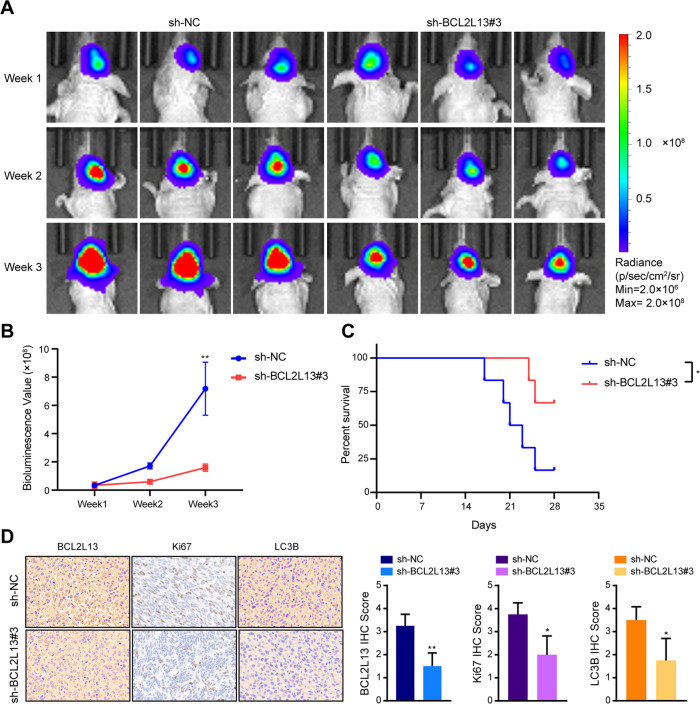
BCL2L13 knockdown inhibits GBM growth in an orthotopic mouse model. **A** GBM#P3 cells expressing luciferase were orthotopically implanted into athymic nude mice, and tumor growth was monitored using the IVIS-200 imaging system for detection of bioluminescence. Bioluminescent signals were measured at days 7, 14, and 21 after implantation. **B** Bioluminescence values to assess tumor growth. **C** Overall survival was determined using Kaplan–Meier survival curves, and a log-rank test was used to assess the statistical significance of the differences. **D** Images and statistical results of immunohistochemical staining for BCL2L13, Ki67, and LC3B in tumors from each group as indicated (scale bars: 50 μm). All data are expressed as the mean ± SD of values from experiments performed in triplicate. *P < 0.05, **P < 0.01, and ***P < 0.001 compared to controls.

To further elucidate the role of BCL2L13 in the development of GBM, an orthotopic mouse model was established with luciferase-stable GBM#P3 cells transduced with sh-NC (*n* = 6) or sh-BCL2L13#3 (*n* = 6), and tumor growth was examined over time using bioluminescence values. The results demonstrated that knockdown of BCL2L13 significantly reduced tumor growth (~55 × 10^8^ vs. ~30 × 10^8^ photons/s, sh-NC vs. sh-BCL2L13; Fig. 8A, B). Kaplan–Meier analysis of the survival data demonstrated a statistically significant difference between sh-NC and sh-BCL2L13 mice (*P* < 0.05, sh-NC vs. sh-BCL2L13; Fig. 8C). Immunohistochemistry (IHC) was then performed on GBM tissue sections from animals to examine proliferation and autophagy. BCL2L13, Ki-67, a marker for proliferation, and LC3B, the marker for autophagy, were decreased in BCL2L13 knockdown GBM cells (Fig. 8D).

## Discussion

This study observed that BCL2L13 expression is higher in glioma than in NBT, according to the data from public databases and our IHC staining results. Moreover, the upregulation of BCL2L13 correlated with tumor histological grades in glioma. Our results also showed that knockdown of BCL2L13 dramatically decreased migration and invasion ability in GBM cells. These data suggested that BCL2L13 aids the malignant progression and aggressive behavior of GBM cells. Based on these findings, BCL2L13 could be identified as a potential target of GBM molecular-targeting therapy.

In the past years, mitochondrial functions are crucial for different cancer cells, including GBM cells. However, this is not surprising, considering that mitochondria are decisive for metabolism and elicit cell death. Mitophagy, a specialized autophagy pathway, ensures the selective removal of damaged or dysfunctional mitochondria and metabolic rewiring, thus supporting the high bioenergetic demand of fast-growing tumor cells. In this study, we revealed that BCL2L13 acts as a critical activator of mitophagy in GBM cells. These data are consistent with another report that indicated that BCL2L13 is a mammalian homolog of the yeast mitophagy receptor Atg32 [22, 23]. However, our results contrast with Anyonya et al., who showed that *BCL2L13* knockdown increased mitophagy in mouse ear mesenchymal stem cells [24], possibly because of differences in cell types. Autophagy has opposed context-dependent cancer roles, and interventions to stimulate and inhibit autophagy have been proposed as cancer therapies [25]. Therefore, it is crucial to understand the role of autophagy in different types of cells after specific treatment. We found that BCL2L13-induced mitophagy contributed to GBM malignant progression. Inhibition of mitophagy by 3-MA or Baf blocked enhanced proliferation, migration, and invasion triggered by BCL2L13 overexpression in GBM cells. These results demonstrated that BCL2L13 induced protective mitophagy in GBM. Highly expressed BCL2L13 in GBM may participate in the resistance processes during radiotherapy, chemotherapy, and target therapy.

Mitochondrial morphology regulated by fusion and fission is essential for maintaining a normal mitochondrial function [26]. In mammalian cells, mitochondrial fusion is regulated by MFN1, MFN2, and OPA1, while the fission of mitochondria is primarily carried out by DNM1L, which translocates from the cytosol to mitochondria, binding to its outer mitochondrial membrane (OMM) partners: MFF, MID49, MID51, and FIS1. Emerging crosstalk has revealed the close links between imbalanced mitochondrial dynamics, mitophagy, and cancers. Mitochondrial fission segregates impaired mitochondria in smaller sizes from the mother mitochondria and may favor mitophagy for eliminating damaged mitochondria [27]. Increased mitochondrial fission has also been reported to promote autophagy, migration, and invasion in ovarian cancer cells in vitro and vivo [28]. In hepatocellular carcinoma cells, increased mitochondrial fission also promotes autophagy and survival [29]. However, the molecular mechanism regulating the dynamic changes of mitochondria is still not fully understood, especially in GBM cells. In this study, BCL2L13 was found to promote mitophagy by increasing mitochondrial fission. Furthermore, we showed that BCL2L13 promotes mitochondrial fission by facilitating DNM1L S616 phosphorylation. Finally, Mdivi-1, a selective mitochondrial fission inhibitor, inhibited mitochondrial fission and mitophagy in BCL2L13 overexpressed cells. These results indicate that BCL2L13 promotes mitochondrial fission-dependent protective mitophagy in GBM cells.

In summary, we demonstrated that BCL2L13 is an oncogene in GBM, which induces mitochondrial fission, thus promoting protective mitophagy. These results suggest that BCL2L13 is a potential therapeutic target in GBM.

## Materials and methods

### Ethics statement

All experimental protocols were approved by the Ethics Committee of the Qilu Hospital (Jinan, China) and performed in accordance with the relevant guidelines and regulations. Written informed consent was obtained from all patients. All animal experiments were approved by the Institutional Animal Care and Use Committee (IACUC) of Shandong University (Jinan, China).

### Database searches

The Cancer Genome Atlas (TCGA, http://cancergenome.nih.gov), Rembrandt (http://www.betastasis.com/glioma/rembrandt), Oncomine (http://www.oncomine.gov), the Human Protein Atlas (http://www.proteinatlas.org), and the Cancer Cell Line Encyclopedia (CCLE, https://portals.broadinstitute.org/ccle) were mined for relevant molecular data.

### Cell lines and cultures

Human glioma cell lines U251, A172, and LN229 were purchased from the Chinese Academy of Sciences Cell Bank (Shanghai, China; TCHu58, TCHu138). NHA, GBM#P3, GBM#BG5, luciferase-stable GBM#P3 were kindly provided by Prof. Rolf Bjerkvig (University of Bergen). U251, A172, LN229, and NHA cells were cultured in Dulbecco modified Eagle medium (DMEM; Thermo Fisher Scientific, SH30022.01B) supplemented with 10% fetal bovine serum (FBS, GE Healthcare Life Sciences, 10082147). GBM#P3 and GBM#BG5 cells were cultured in Neurobasal Medium (Thermo Fisher Scientific; Waltham, MA, USA) containing B27 supplement (20 μL/mL), FGF (20 ng/mL) and EGF (20 ng/mL) for cell culture and passage. Culturing medium of GBM#P3 and GBM#BG5 cells were changed into DMEM supplemented with 10% FBS for cell plating and further functional experiments. Cells were maintained at 37 °C in a humidified chamber containing 5% CO_2_.

### *BCL2L13* silencing and overexpression

Lentiviral vectors expressing human shRNA targeting *BCL2L13* (sh-BCL2L13#1: 5’‐GGA AGA GAG CCU UGU GGA ATT‐3’; sh-BCL2L13#2: 5’‐GGA CAA AGA AAU UUC UGA ATT‐3’; sh-BCL2L13#3: 5’‐CUG CAG AAG AUA GCA AUG ATT‐3’, GenePharma Shanghai) or scrambled‐control (sh-NC) were used to generate stable cell clones expressing sh-BCL2L13 or a nonspecific shRNA as the control. Cells were plated and infected with lentiviruses expressing sh-NC/BCL2L13 for 24 h, according to the manufacturer’s protocol. Cells were transfected with pENTER-BCL2L13 to induce the overexpression of BCL2L13 and with an empty pENTER vector as a control. Transfection was performed with Lipofectamine 3000 (Life Technologies, Carlsbad, CA). Transfected clones were selected using 1 mg/mL of puromycin (Selleckchem). qRT-PCR and western blotting analysis were used to evaluate shRNA knockdown or overexpression efficiency.

### Quantitative real-time PCR (qRT-PCR)

Total RNA was prepared from treated cells using Trizol (Thermo Fisher Scientific; MA, USA). Briefly, after centrifugation, the aqueous layer was transferred to a new Eppendorf tube, and isopropanol was added to precipitate total RNA. cDNA was generated from total RNA using the ReverTra Ace qPCR RT Kit (TOYOBO; Osaka, Japan). qRT-PCR was performed with SYBR Green Master (Roche; Basel, Switzerland) on the 480II Real Time PCR Detection System (Roche; Basel, Switzerland). GAPDH mRNA was used to normalize mRNA expression. The results are representative of at least three independent experiments. The sequences of the PCR primers used are the following: GAPDH-F 5’-GCACCGTCAAGGCTGAGAAC-3’, R 5’- TGGTGAAGACGCCAGTGGA-3’; BCL2L13-F 5’-AGGACTATTCGGCAGAGTACAT-3’, R 5’-TGATTCCAGGGTATTCCTCCTC-3’.

### Western blot analysis

Cell lysates (20 µg protein) were subjected to western blot analysis, according to previously described protocols. Membranes were incubated with the following antibodies from Cell Signaling Technology: DNM1L (8570), phospho616-DNM1L (4494), phospho637-DNM1L (6319), MFN1 (14739), BECN1 (3495), MAP1LC3B (12741), GAPDH (5174). Additional antibodies were BCL2L13 (Proteintech, 16612-1-AP) and FIS1 (Abcam, ab156865).

### Wound-healing assay

Cells in the logarithmic phase were put on a six‐well plate and cultured for 24 h until 90% confluence. After making a vertical wound on the board’s surface with the tip of a P200 pipette, cells were washed several times with PBS to remove cell debris. Then cells were cultured in a serum-free medium. Cell positions were recorded, and migrating rates were measured at 0 and 24 h.

### Transwell assay

Matrigel (BD Biosciences, NJ) was obtained to cover the bottom membrane of transwell chambers (24 holes, Corning Inc., NY) and measure cells’ invasive ability. Each transwell membrane enclosed a mixture of Matrigel and medium at the proportion of 1:2 at 50 µl. The upper chamber was inoculated with 2.5 × 10^4^ cells, while the serum, growth factors, and chemokines were placed into the lower chamber and cultured in 5% CO_2_ at 37 °C for 24 h. Then, chambers were stabilized with paraformaldehyde for 10 min, and 500 µl 0.1% crystal violet was added for 10 min before being washed out. After air-drying, the stained cells were photographed and counted under the light microscope (×200) in four randomly selected fields.

### 3D Invasion

Cell spheres that were cultured in low-attachment U-bottom 96-well plates were embedded in Matrigel (Trevigen; Gaithersburg, MD, USA) and cultured for 36 h (GBM#P3) or 120 h (GBM#BG5) at 37 °C for the 3D tumor spheroid invasion experiment. The sphere’s diameter was considered the starting point for quantification.

### TEM

Cells were fixed with 4% glutaraldehyde and post-fixed with 1% OsO_4_ in 0.1 M cacodylate buffer for 2 h. The samples were then stained with 1% Millipore-filtered uranyl acetate, dehydrated in increasing ethanol concentrations, and infiltrated and embedded in epoxy resin (ZXBR, Spon 812). Electron photomicrographs were taken from GBM cells’ ultrastructures using a transmission electron microscope (JEM-1200EX II, JEOL; Tokyo, Japan).

### GFP-LC3B transfection and evaluation

GFP-LC3B (pBABEpuro, 22405)-expressing vectors were obtained from Addgene and deposited by EndoFree Plasmid Maxi Kit (QIAGEN, 12362). Lentiviral supernatants were prepared according to the manufacturer’s instructions and provided by GenePharma. Lentiviral infections were performed accordingly. GFP-LC3B stable GBM cells were fluorescently labeled with 25 nM MitoTracker Red (Invitrogen, Molecular Probes) for mitochondrial morphology. Cells were viewed using an SP8 confocal microscope (Leica, Germany). All images were analyzed by ImageJ software (MD, USA). Autophagy/mitophagy was quantified by calculating the average number of GFP-LC3B puncta/GFP-LC3B, and RFP-mito merged puncta per cell in five high-power fields.

### Detection of in vitro mitophagy using mt-Keima fluorescent reporter

Imaging of mt-Keima GBM#P3, GBM#BG5 and A172 cells was performed according to the instruction, using different settings for GFP and red fluorescent protein (RFP). mt-Keima is a ratiometric pH-sensitive fluorescent protein that exhibits green fluorescence (excitation 440 nm) in basic or neutral conditions and red fluorescence (excitation 550 nm) in acidic conditions. For our experiments, the settings used were green channel (excitation 440 nm, emission 570–695 nm) to visualize normal mitochondria, and red channel (excitation 550 nm, emission 570–695 nm) to visualize mitochondria undergoing mitophagy. The mt-Keima GBM#P3, GBM#BG5 and A172 cells were treated as previously mentioned, followed by confocal imaging. Data were quantified, using ImageJ software, as the total number of red pixels divided by the total number of green pixels.

### Cell counting kit (CCK)-8 assay

Cell viability was assessed with the cell counting kit-8 (CCK-8; Dojindo, CK04-500). Cells (1.0 × 10^4^ cells/well) were seeded into 96-well plates and incubated at 37 °C overnight. 3-MA (Sigma-Aldrich, M9281) or Baf (Sigma-Aldrich, B1793) were dissolved in DMSO (Sigma-Aldrich, D2650) and diluted to working concentrations in a culture medium. After the desired treatment, cells were incubated for an additional 4 h at 37 °C with 10 μL of CCK-8 in 100 μL of serum-free DMEM. The absorbance at 450 nm was measured using a microplate reader (Bio-Rad, model 680; Hercules, CA, USA).

### Intracranial xenograft model

Athymic mice (male; 4 weeks old; ~22 g) were provided by Shanghai SLAC Laboratory Animal Co., Ltd (Shanghai, China). The mice were anesthetized with chloral hydrate and secured on a stereotactic frame. A longitudinal incision was made in the scalp, and a 1 mm-diameter hole was drilled 2.5 mm lateral to the bregma. Luciferase-stable GBM#P3 glioma cells (2 × 10^5^) in 20 μL of serum-free DMEM were implanted 2.5 mm into the right striatum using a Hamilton syringe. Mice were monitored by bioluminescence imaging every week. Briefly, mice were injected with 100 mg luciferin (Caliper, 122796), simultaneously anesthetized with isoflurane, and subsequently imaged with a cooled charge-coupled device camera (IVIS-200, Xenogen; Alameda, CA, USA). Bioluminescence values of tumors were quantified using the Living Image 2.5 software package (Xenogen). Mice were euthanized after 28 days and perfused with 4% paraformaldehyde in PBS. Brains were coronally sectioned for immunohistochemistry assays.

### Immunohistochemistry

Paraffin-embedded samples were sectioned (4 µm) and mounted on microscopic slides. Heat-induced epitope retrieval was performed in 10 mmol/L citric acid buffer at pH 7.2 in a microwave. Sections were incubated with the primary antibody at 4 °C overnight (LC3B, 1:200, Cell Signaling Technology, 12741 S; Ki67, 1:200, Cell Signaling Technology, 9027; BCL2L13, 1:20, Proteintech 16612-1-AP), rinsed with PBS, and incubated with horseradish peroxidase-linked goat anti-rabbit secondary antibody (ZSGB-BIO, PV-9000). Visualization was achieved using diaminobenzidine (ZSGB-BIO, ZLI-9033) as the substrate, and slides were counterstained with Mayer hematoxylin (Beyotime Biotechnology, C0107).

### Statistical analysis

Three independent experiments were performed, and results were expressed as the mean ± the standard deviation (SD). Data were compared using paired Student *t* tests for two-group comparison and one-way analysis of variance (ANOVA) for multi-group comparisons in GraphPad Prism 8 software (San Diego, CA, USA). Kaplan–Meier survival curves were generated and compared using the log-rank test. *P* values determined from different comparisons <0.05 were considered statistically significant and are indicated as follows: *P < 0.05; ** *P* < 0.01; ***P < 0.001.

### Reporting summary

Further information on research design is available in the Nature Research Reporting Summary linked to this article.

## Supplementary information

SUPPLEMENTAL Figure 1

SUPPLEMENTAL Figure 2

SUPPLEMENTAL Figure 3

Reporting Summary

Supplementary figure legends

Full and uncropped western blots.pdf

## Data Availability

Datasets and other files generated, analyzed, or used during this study are available from the corresponding author upon reasonable request.
