# Supplementary figures and images for: BCL2L13 promotes mitophagy through DNM1L-mediated mitochondrial fission in glioblastoma
Source: Cell Death Dis. 2023 Sep 2;14(9):585. doi: 10.1038/s41419-023-06112-4 (PMC10475114; doi:10.1038/s41419-023-06112-4)

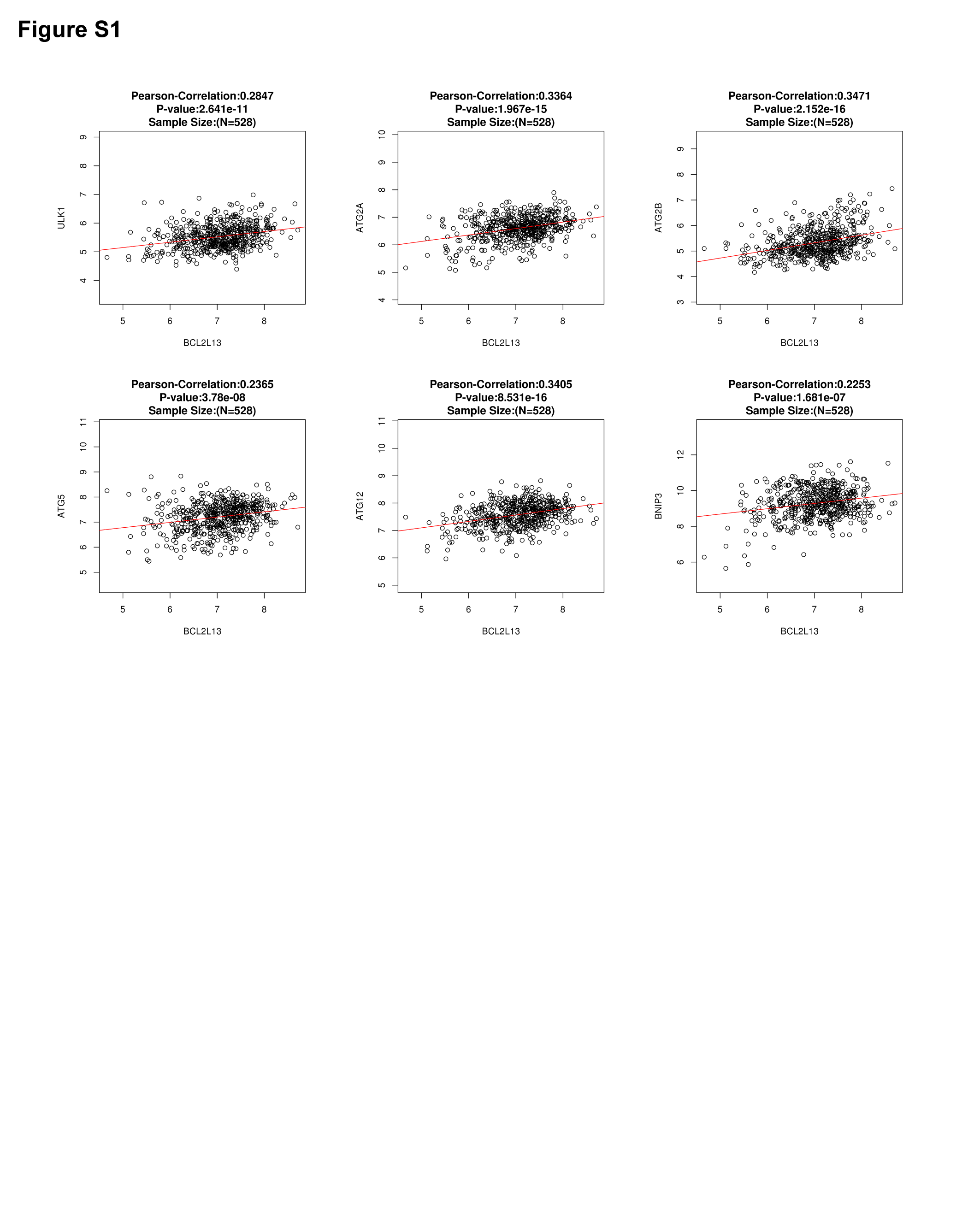

Supplement: Supplementary file 1 — SUPPLEMENTAL Figure 1 [file 41419_2023_6112_MOESM1_ESM.tif]

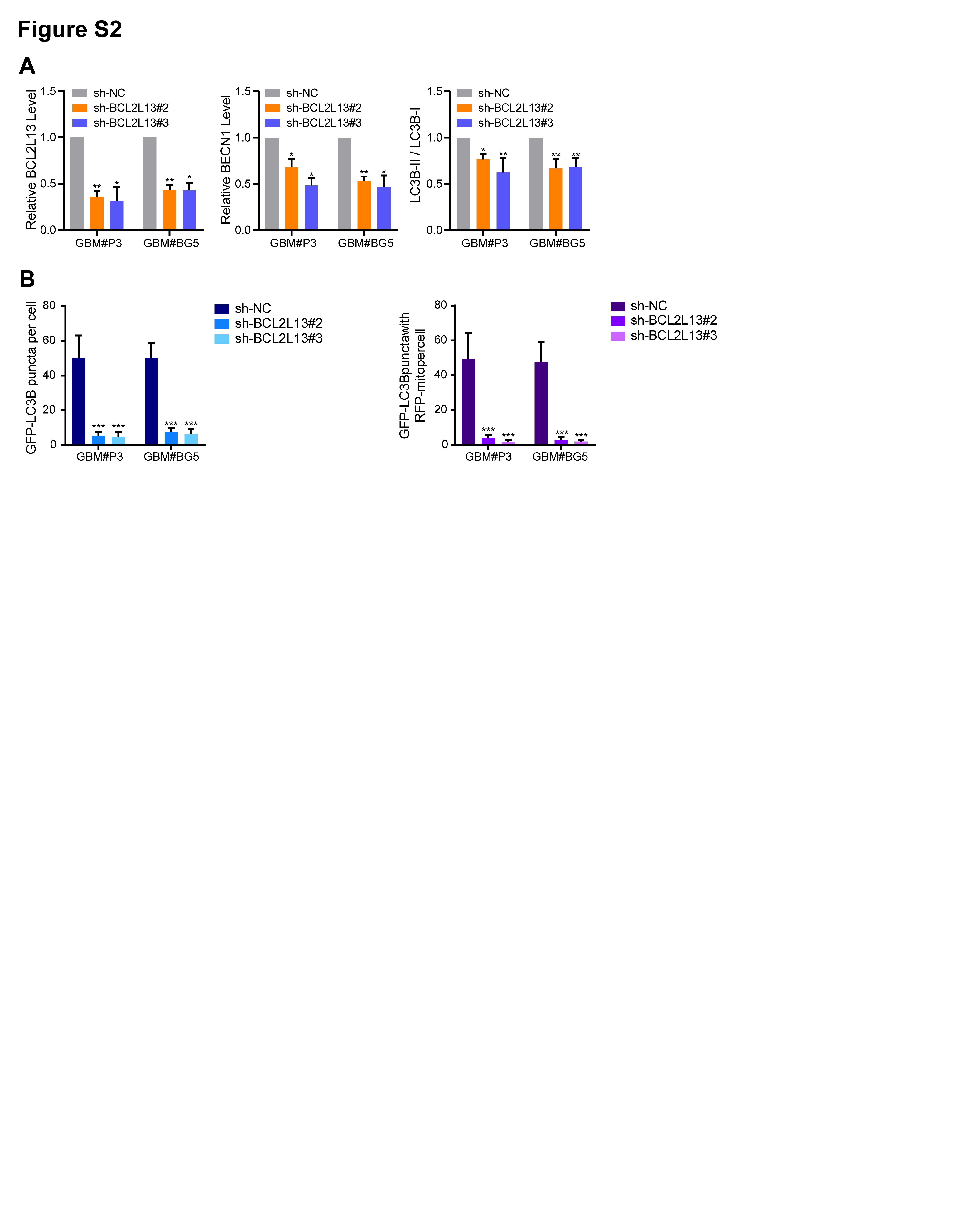

Supplement: Supplementary file 2 — SUPPLEMENTAL Figure 2 [file 41419_2023_6112_MOESM2_ESM.tif]

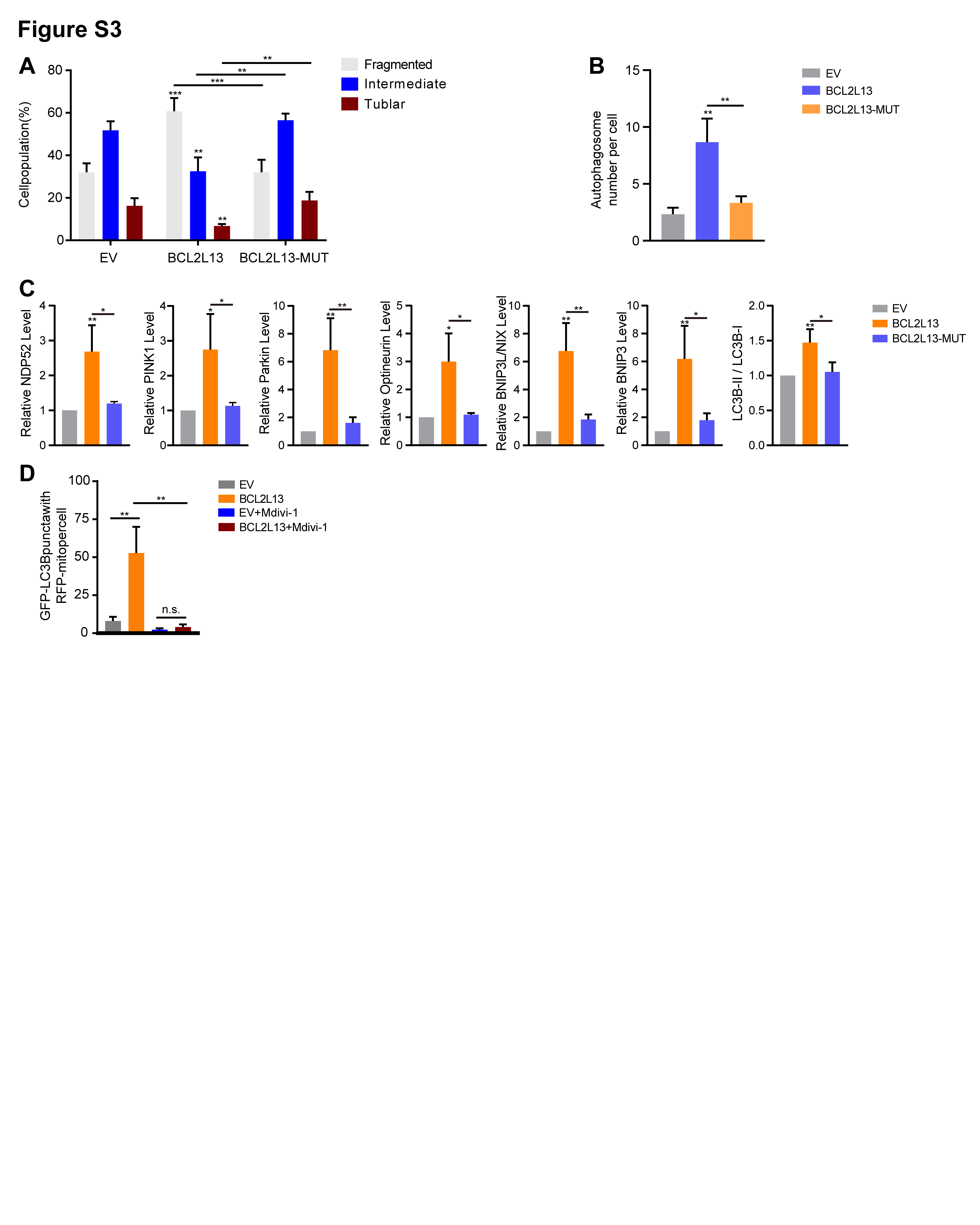

Supplement: Supplementary file 3 — SUPPLEMENTAL Figure 3 [file 41419_2023_6112_MOESM3_ESM.tif]
