# Supplementary material for: BCL2L13 promotes mitophagy through DNM1L-mediated mitochondrial fission in glioblastoma
Source: Cell Death Dis. 2023 Sep 2;14(9):585. doi: 10.1038/s41419-023-06112-4 (PMC10475114; doi:10.1038/s41419-023-06112-4)
Supplement: Supplementary file 5 — Supplementary figure legends [file 41419_2023_6112_MOESM5_ESM.docx]

**Supplementary figure legends**

**Figure S1** Correlation between BCL2L13 and autophagy-related genes in gliomas determined using TCGA dataset.

**Figure S2A** Quantification of the proteins level of GBM#P3 and GBM#BG5 cells after transfected with sh-NC, sh-BCL2L13#2 and sh-BCL2L13#2.

**Figure S2B** Quantification of autophagosomes in sh-NC/BCL2L13 GBM cells. **b-c** Quantification of GFP-LC3B puncta, GFP-LC3B and RFP-mito colocalization puncta in sh-NC/BCL2L13 GBM cells.

**Figure S3A** Quantification of mitochondrial morphology population in A172-EV/BCL2L13/BCL2L13-MUT cells.

**Figure S3B** Quantification of autophagosomes in A172-EV/BCL2L13/BCL2L13-MUT cells.

**Figure S3C** Quantification of the proteins level of A172 cells after transfected with EV/BCL2L13/BCL2L13-MUT.

**Figure S3D** Quantification of GFP-LC3B and RFP-mito colocalization puncta in GBM cells A172-EV/BCL2L13 cells after Mdivi-1 treatment.
