# Supplementary material for: BCL2L13 promotes mitophagy through DNM1L-mediated mitochondrial fission in glioblastoma
Source: Cell Death Dis. 2023 Sep 2;14(9):585. doi: 10.1038/s41419-023-06112-4 (PMC10475114; doi:10.1038/s41419-023-06112-4)
Supplement: Supplementary file 6 — Full and uncropped western blots.pdf [file 41419_2023_6112_MOESM6_ESM.pdf]

# Figure 1

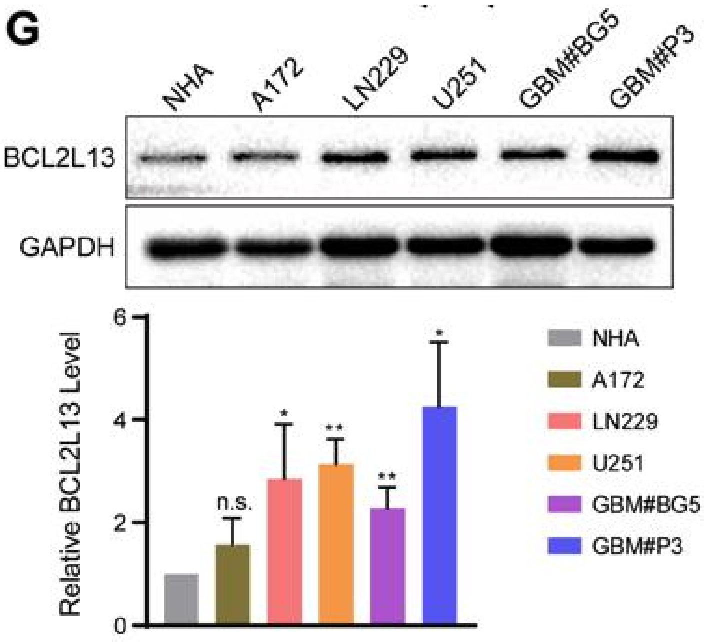
G
NHA
A172
LN229
U251
GBM#BG5
GBM#P3
BCL2L13
GAPDH
Relative BCL2L13 Level
6
4
2
0
n.s.
*
**
**
*
NHA
A172
LN229
U251
GBM#BG5
GBM#P3

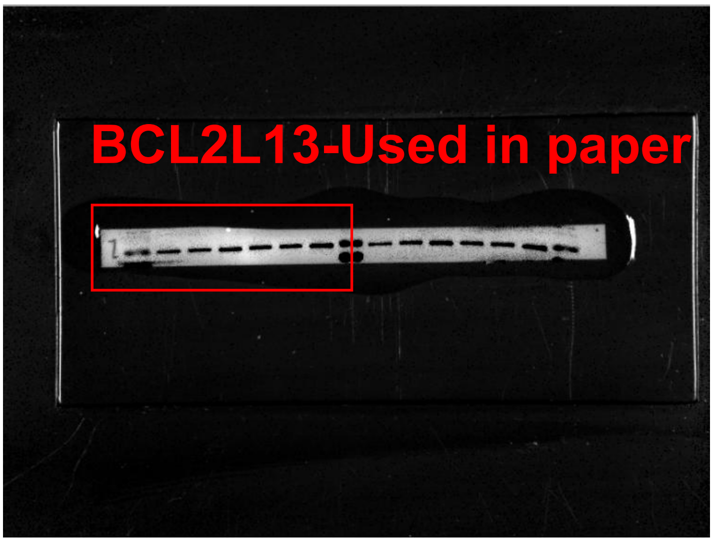
BCL2L13-Used in paper

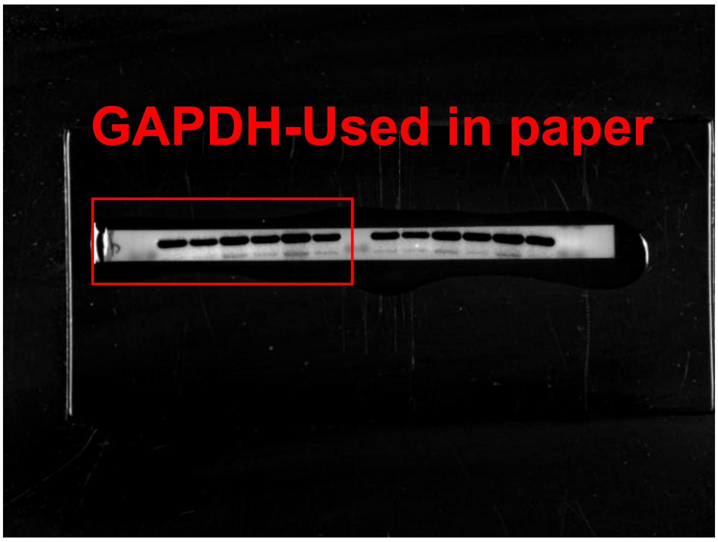
GAPDH-Used in paper

# Figure 2

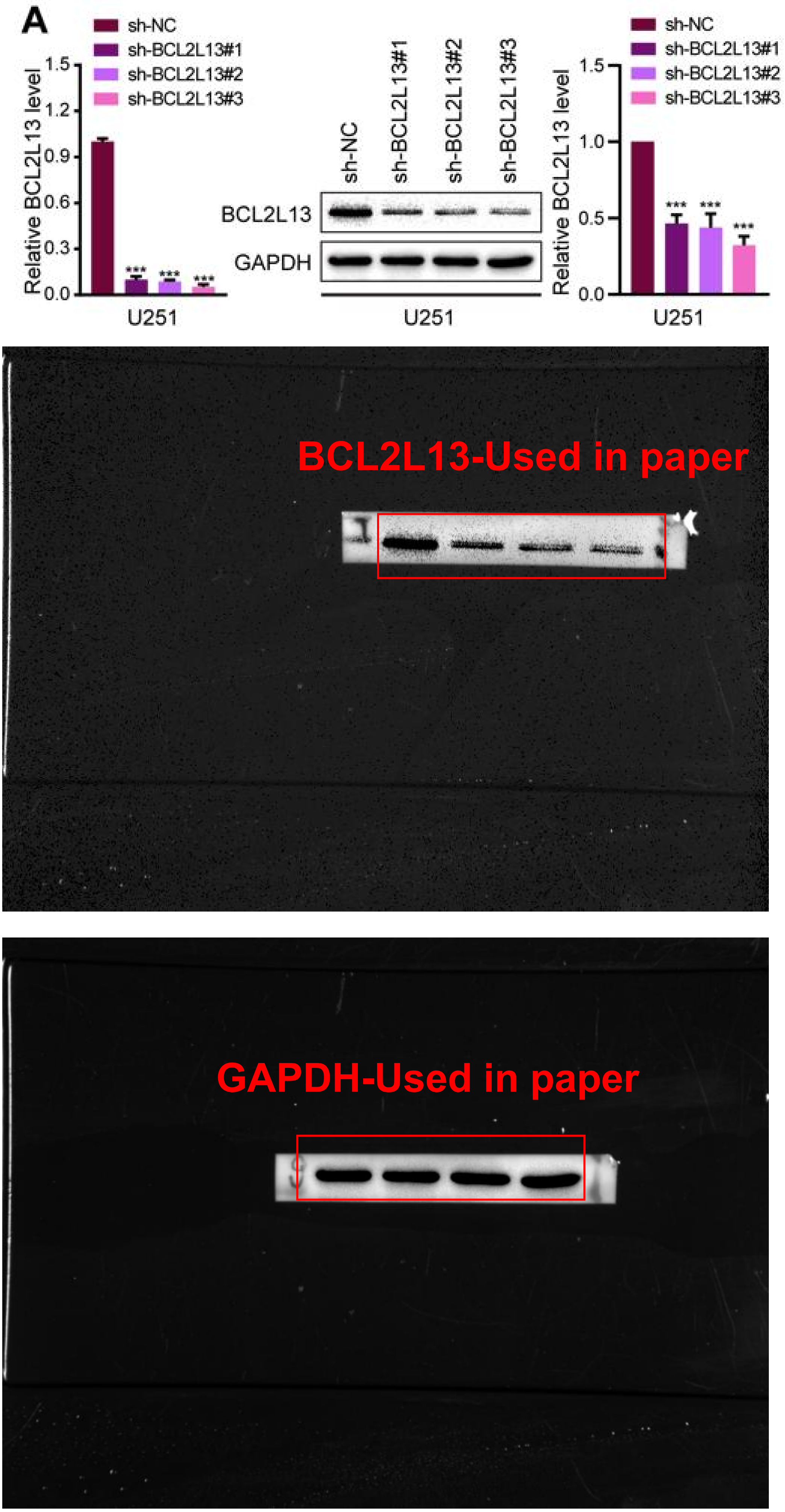
A
sh-NC
sh-BCL2L13#1
sh-BCL2L13#2
sh-BCL2L13#3
Relative BCL2L13 level
1.5
1.2
0.9
0.6
0.3
0.0
***
U251
sh-NC
sh-BCL2L13#1
sh-BCL2L13#2
sh-BCL2L13#3
BCL2L13
GAPDH
U251
Relative BCL2L13 level
1.5
1.0
0.5
0.0
***
U251
BCL2L13-Used in paper
GAPDH-Used in paper

# Figure 2

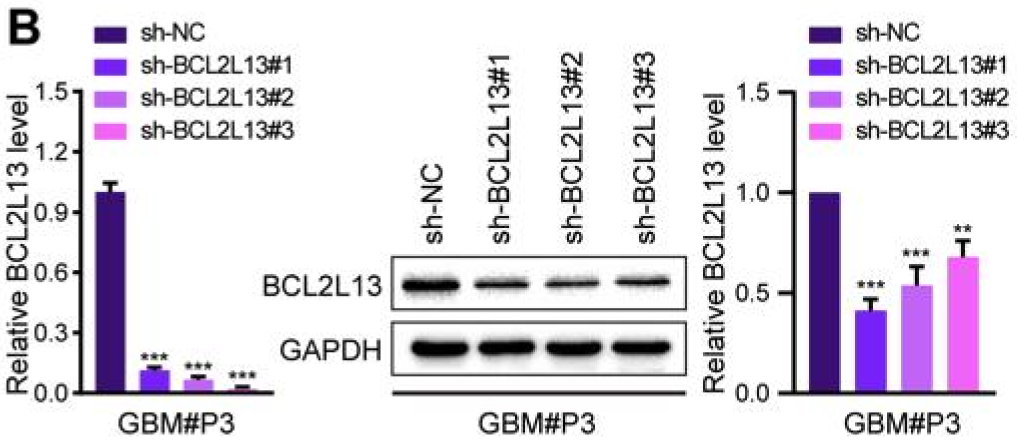
B
sh-NC
sh-BCL2L13#1
sh-BCL2L13#2
sh-BCL2L13#3
Relative BCL2L13 level
1.5
1.2
0.9
0.6
0.3
0.0
***
***
***
GBM#P3
sh-NC
sh-BCL2L13#1
sh-BCL2L13#2
sh-BCL2L13#3
BCL2L13
GAPDH
GBM#P3
sh-NC
sh-BCL2L13#1
sh-BCL2L13#2
sh-BCL2L13#3
Relative BCL2L13 level
1.5
1.0
0.5
0.0
***
***
**
GBM#P3

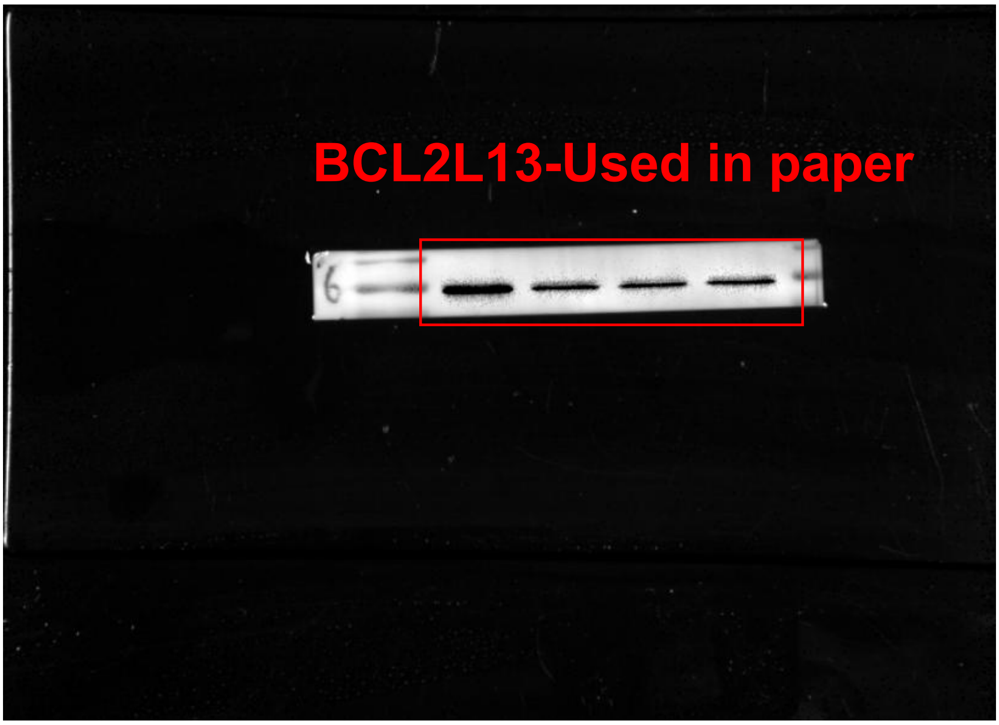
BCL2L13-Used in paper

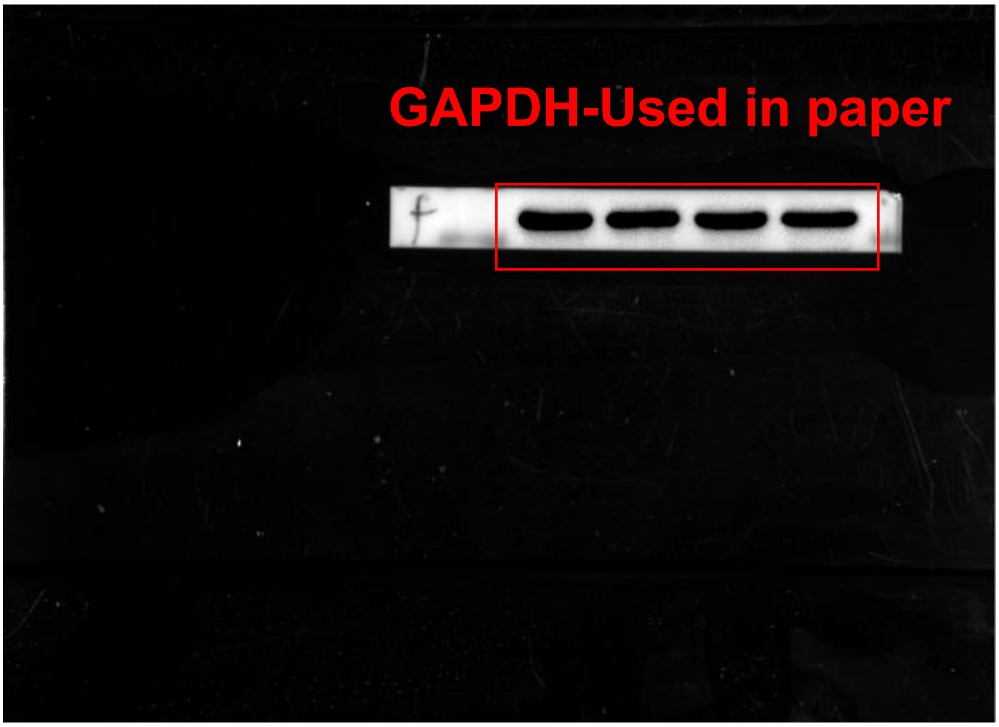
GAPDH-Used in paper

# Figure 2

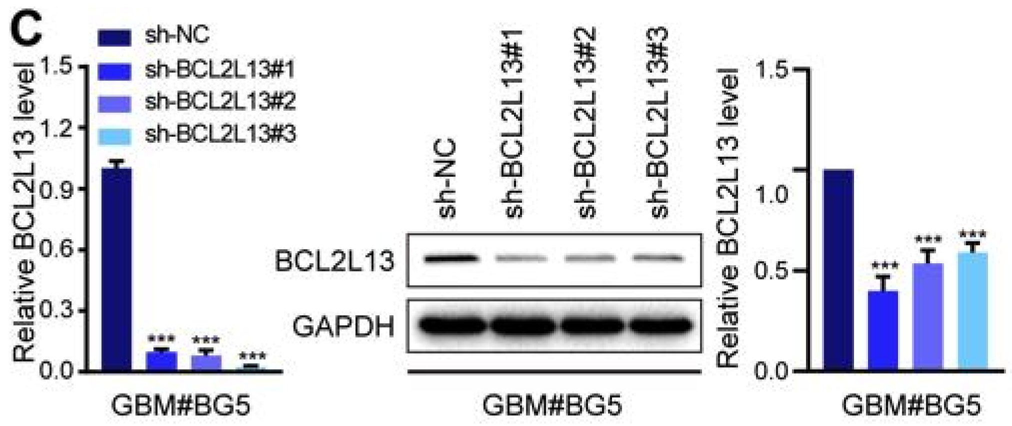
C
sh-NC
sh-BCL2L13#1
sh-BCL2L13#2
sh-BCL2L13#3
Relative BCL2L13 level
1.5
1.2
0.9
0.6
0.3
0.0
***
GBM#BG5
sh-NC
sh-BCL2L13#1
sh-BCL2L13#2
sh-BCL2L13#3
BCL2L13
GAPDH
GBM#BG5
Relative BCL2L13 level
1.5
1.0
0.5
0.0
GBM#BG5

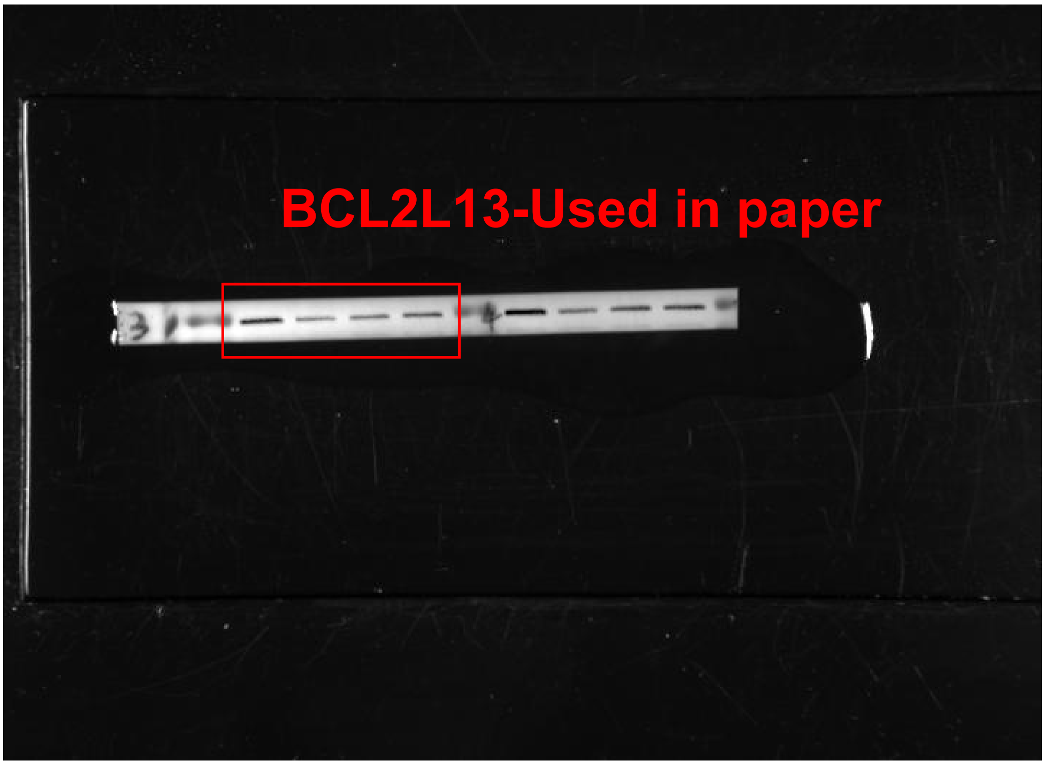
BCL2L13-Used in paper

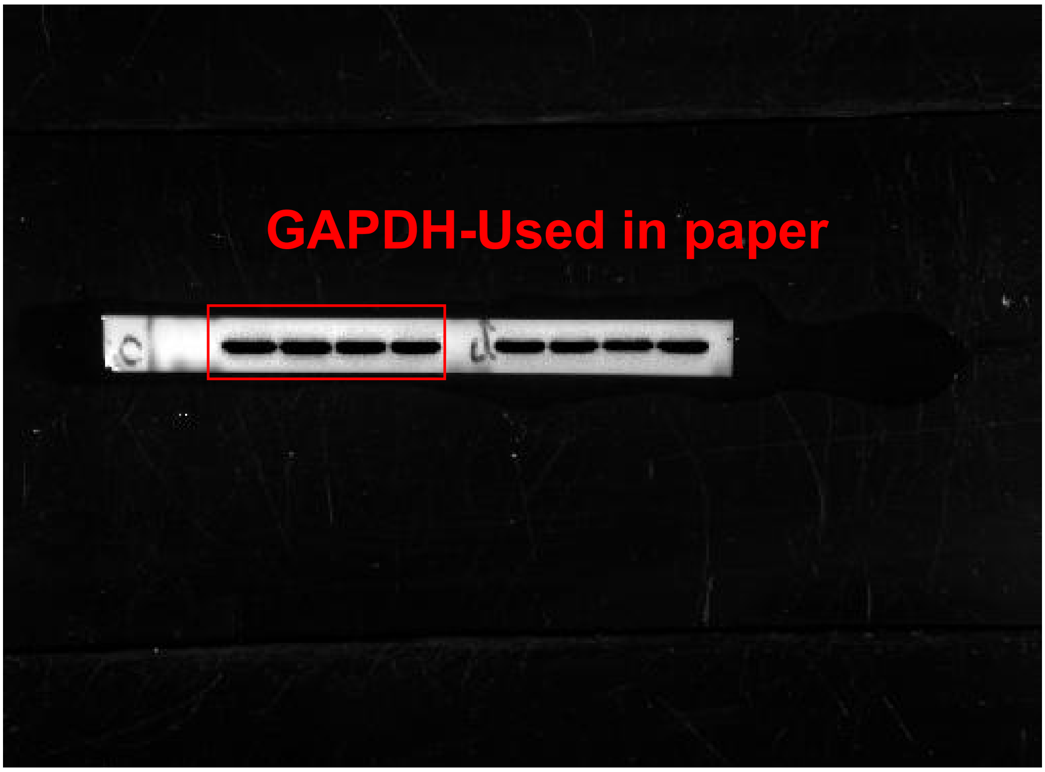
GAPDH-Used in paper

# Figure 4

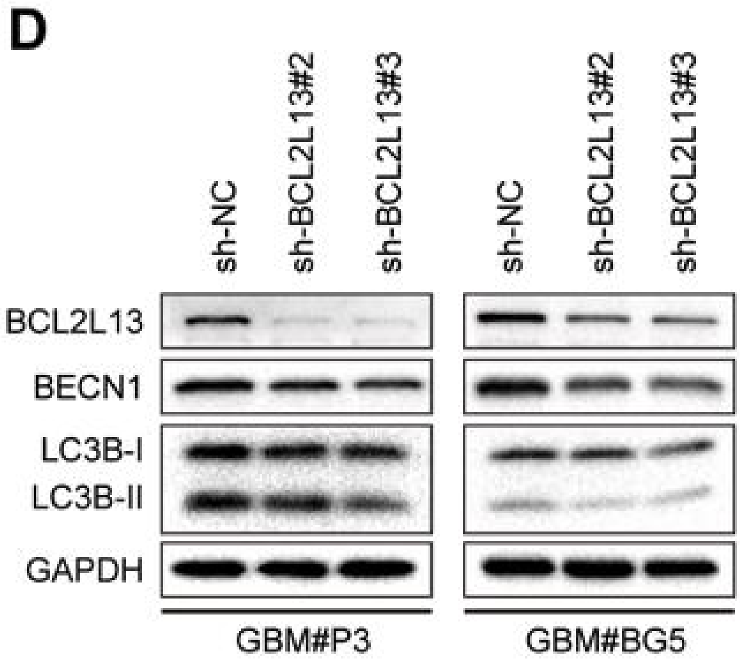


GBM#P3

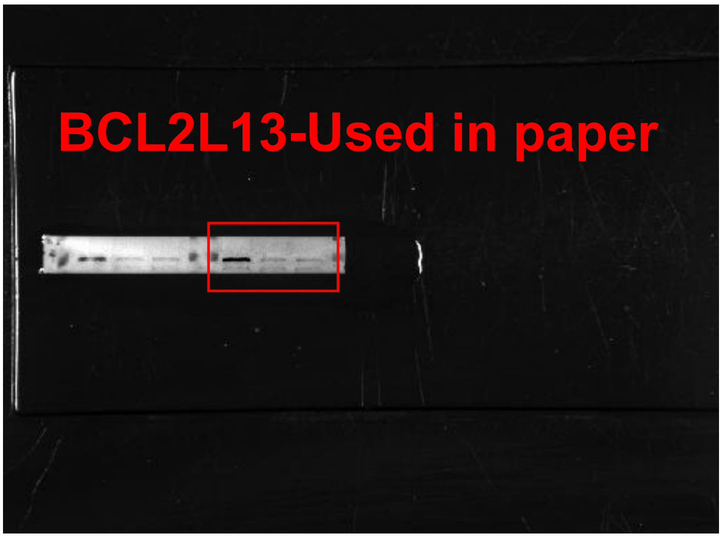


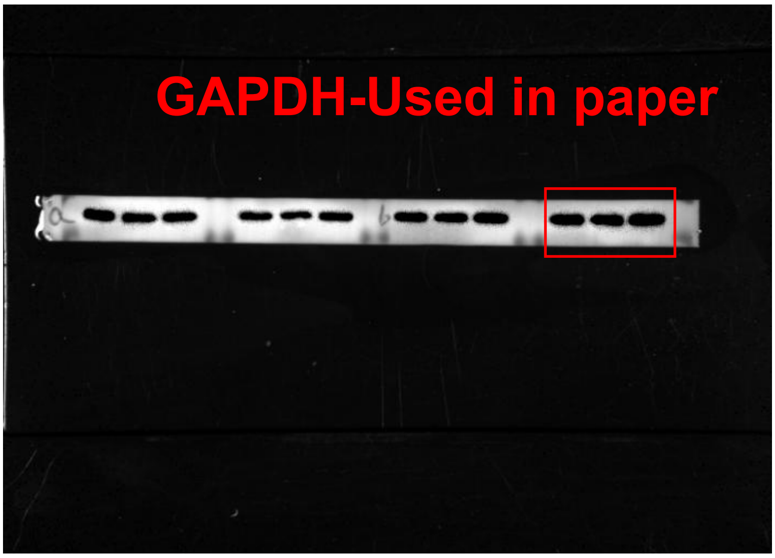


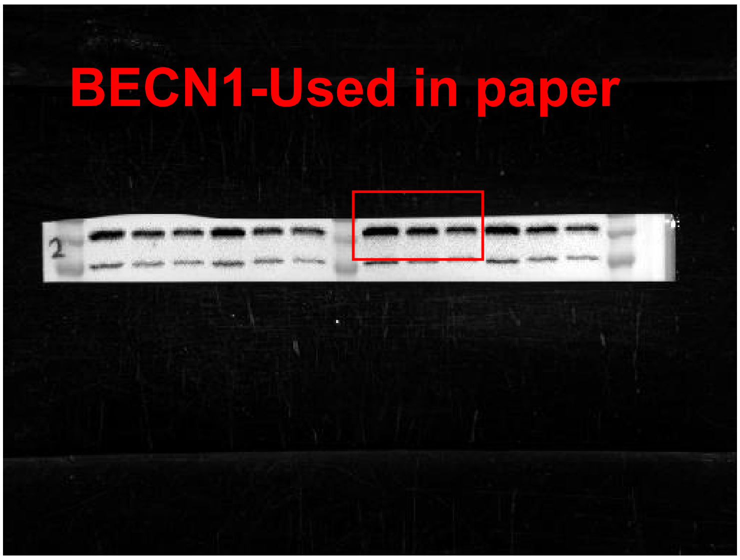


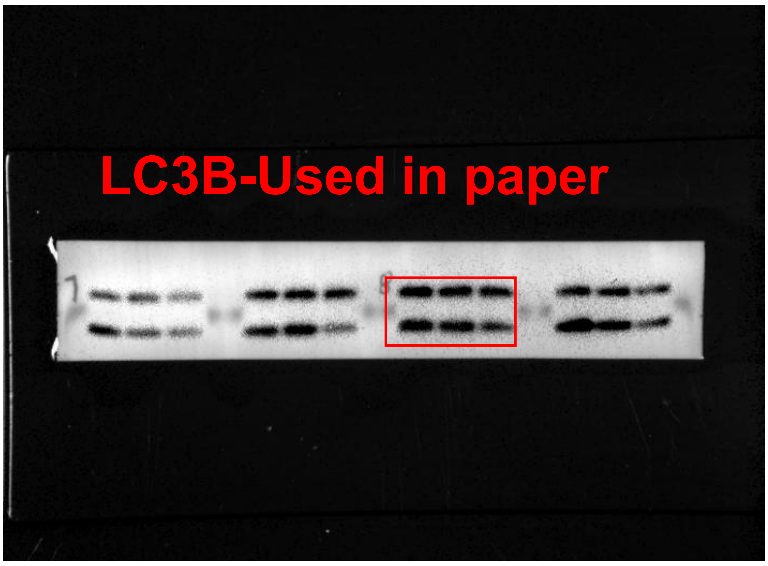

# Figure 4

**D**

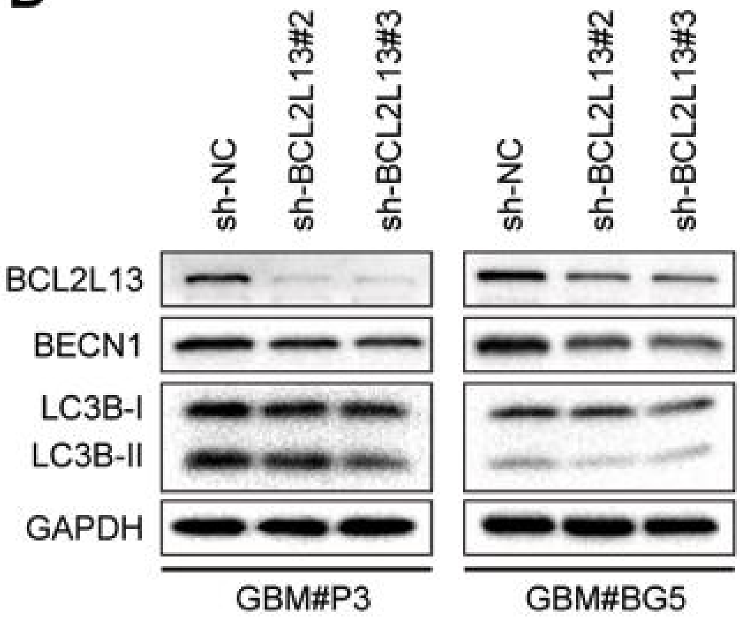


## GBM#BG5

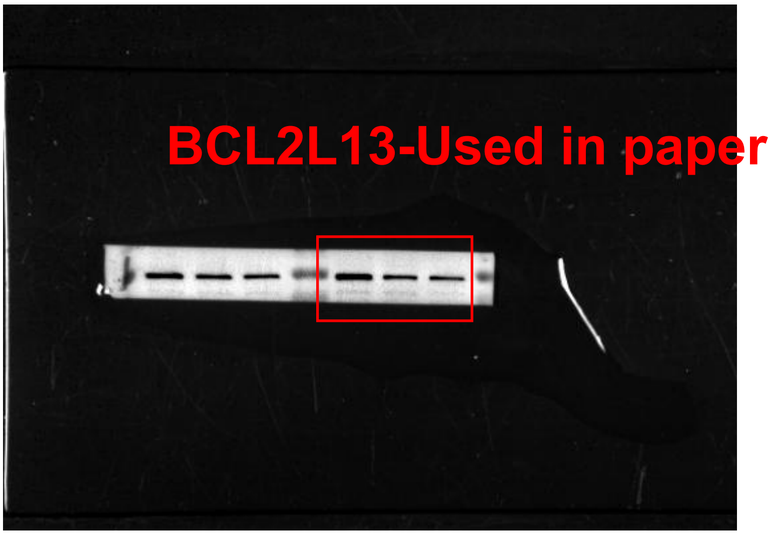


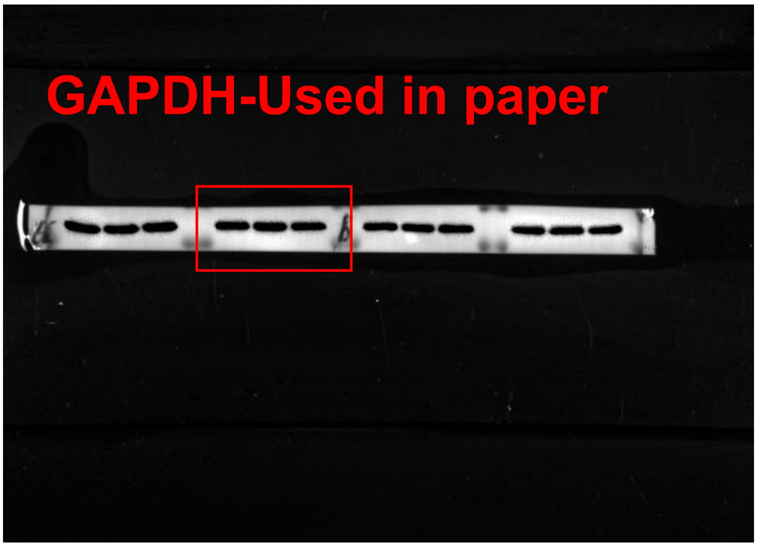


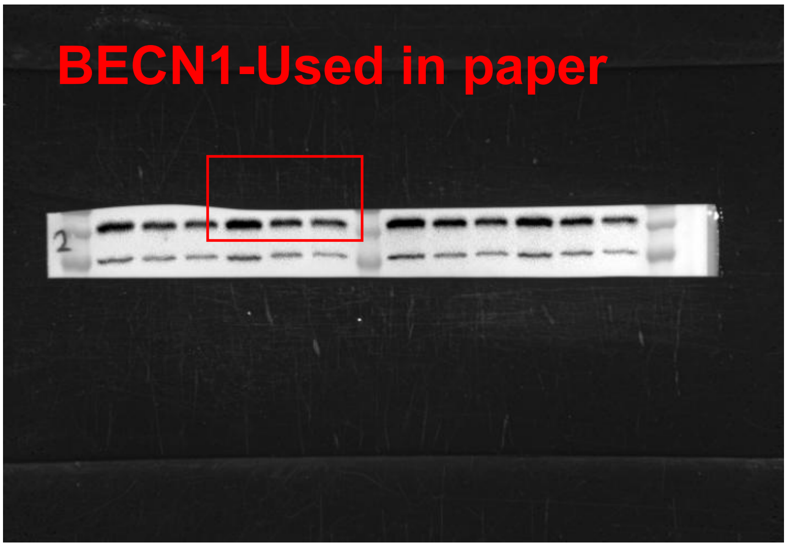


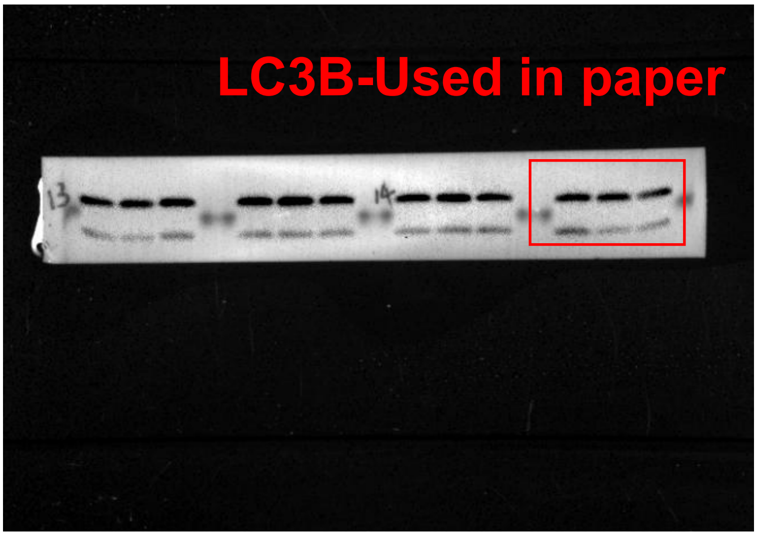

# Figure 4

G

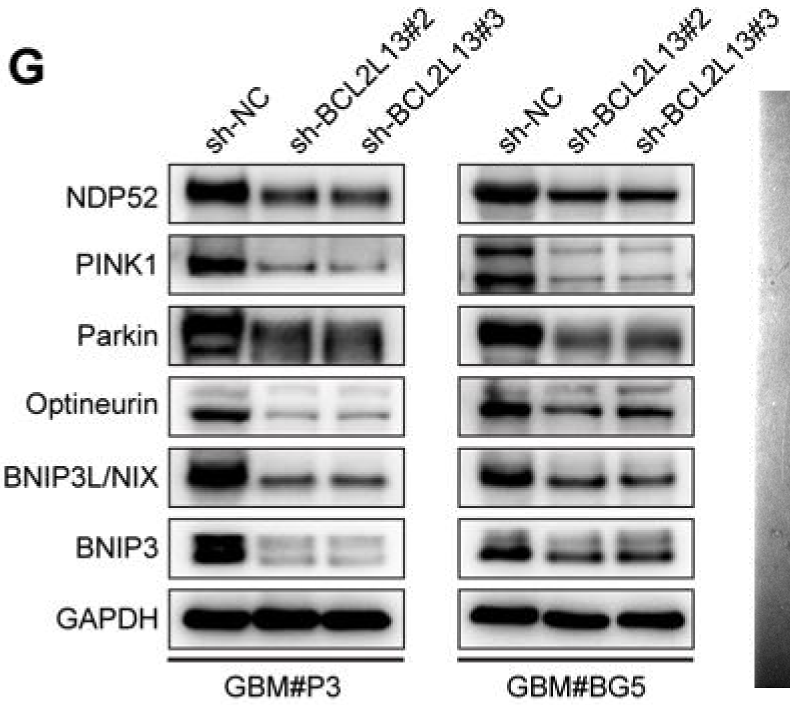


GBM#P3

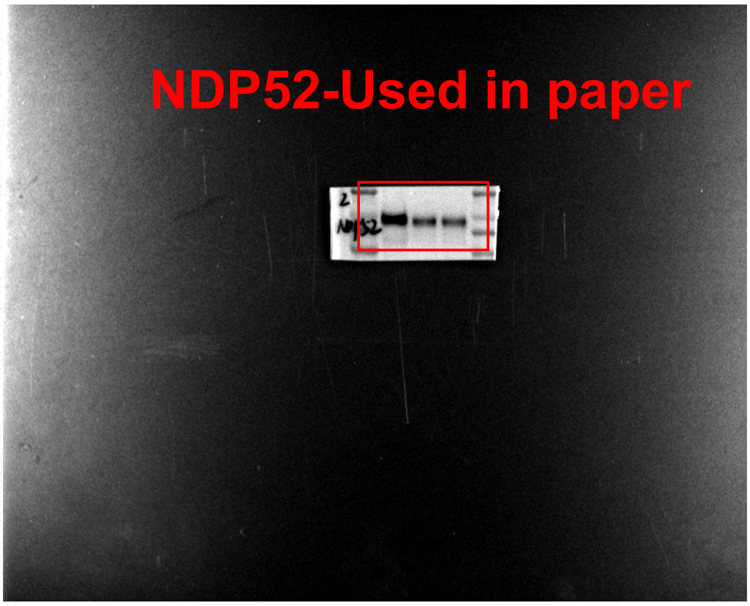


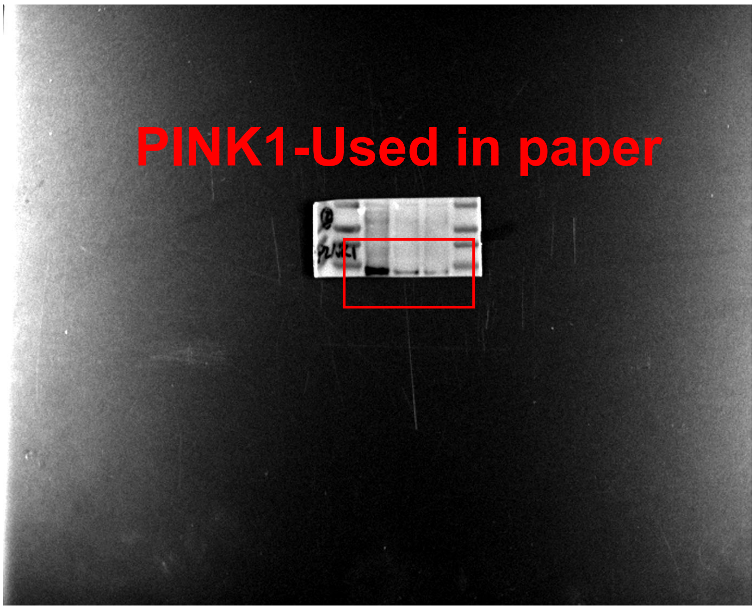


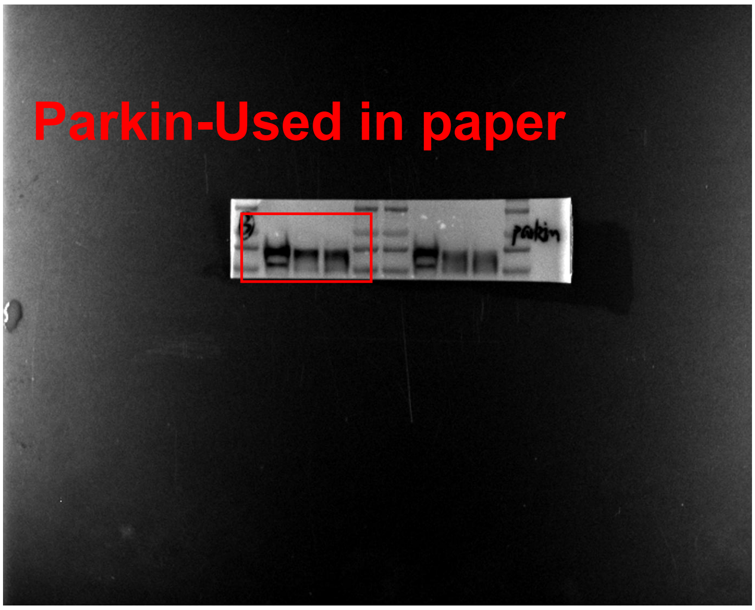


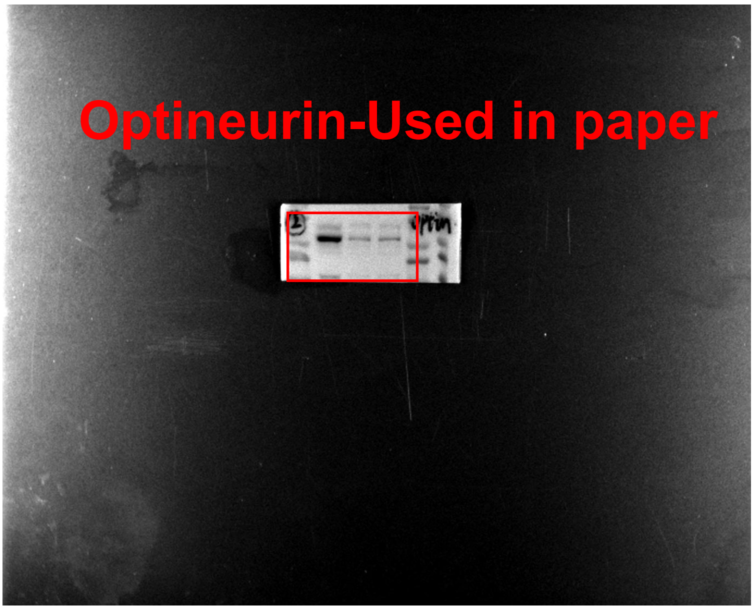


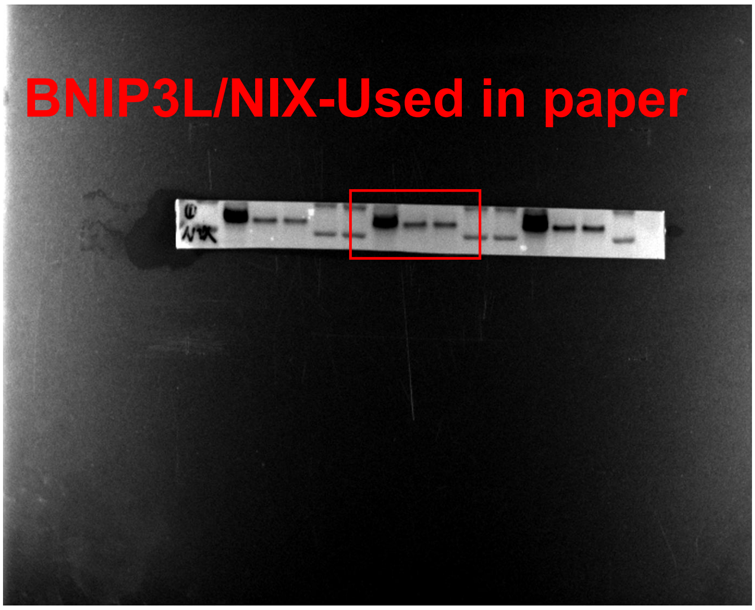

# Figure 4

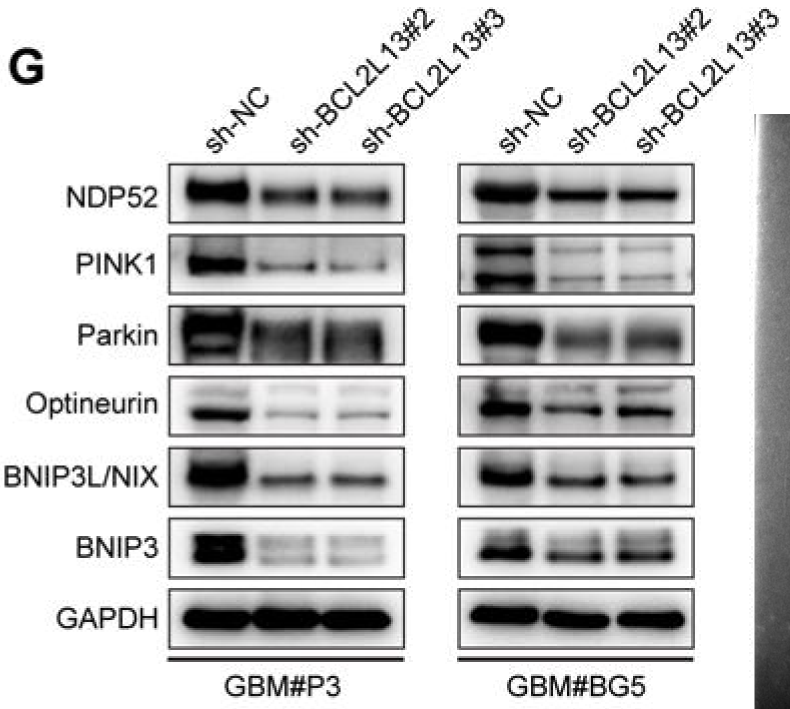


GBM#P3

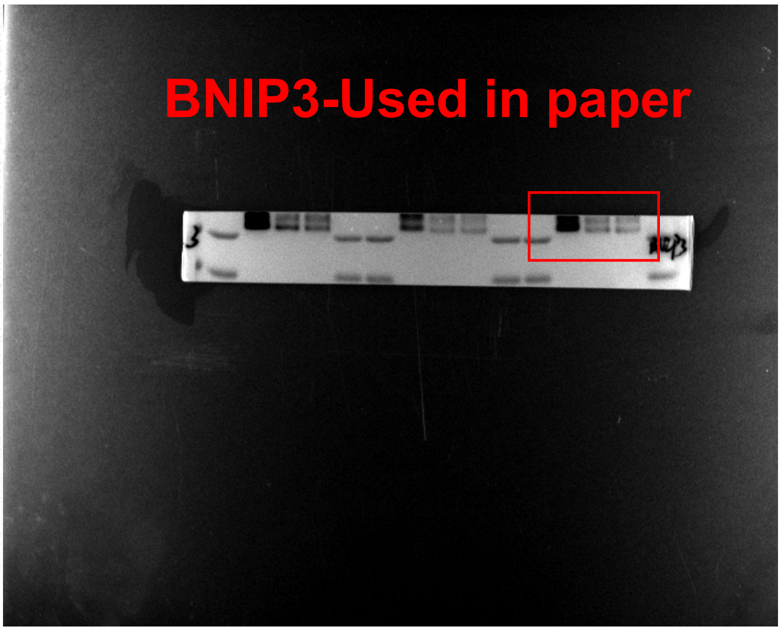


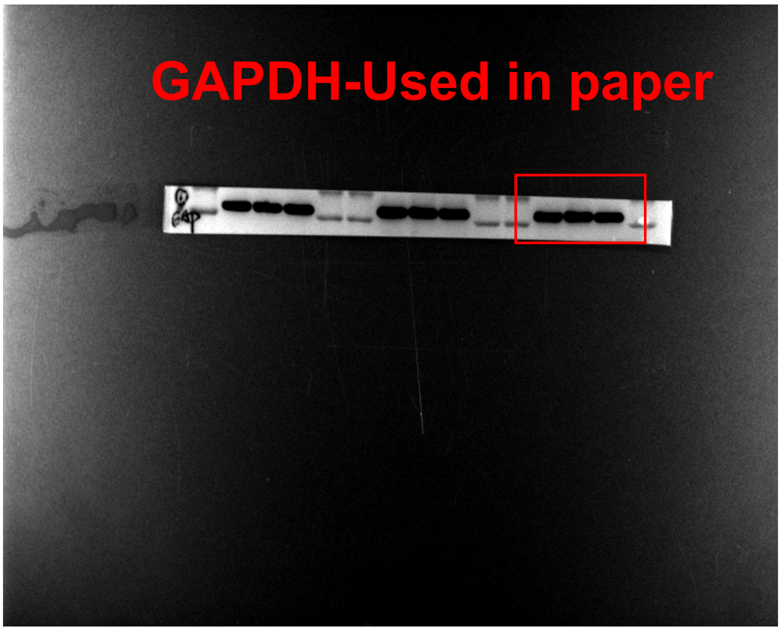

# Figure 4

GBM#BG5

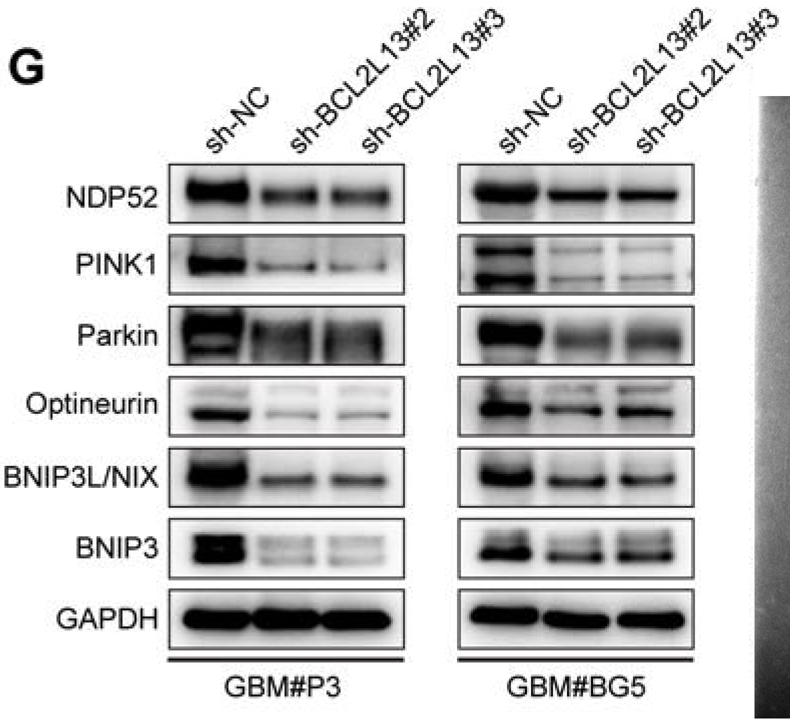


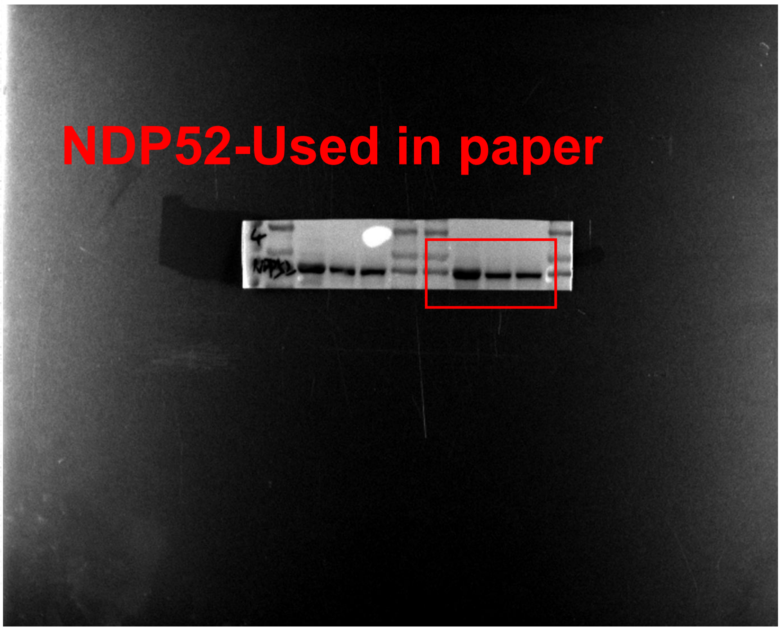


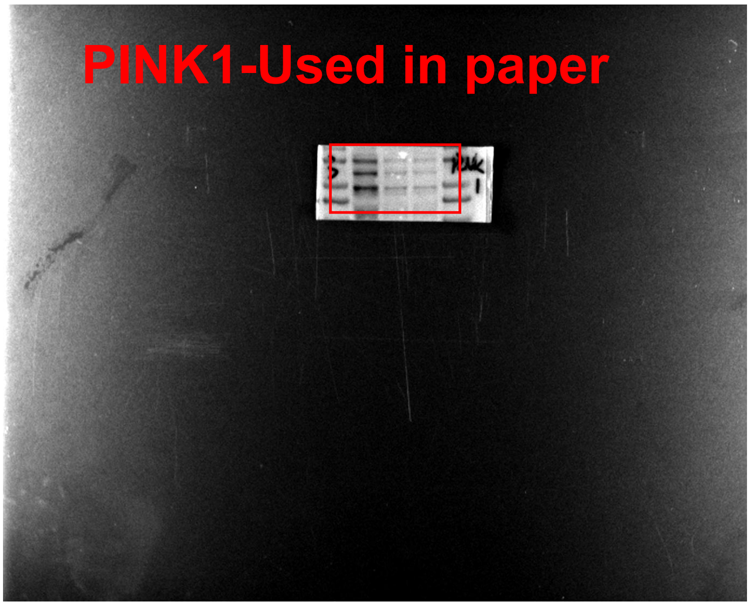


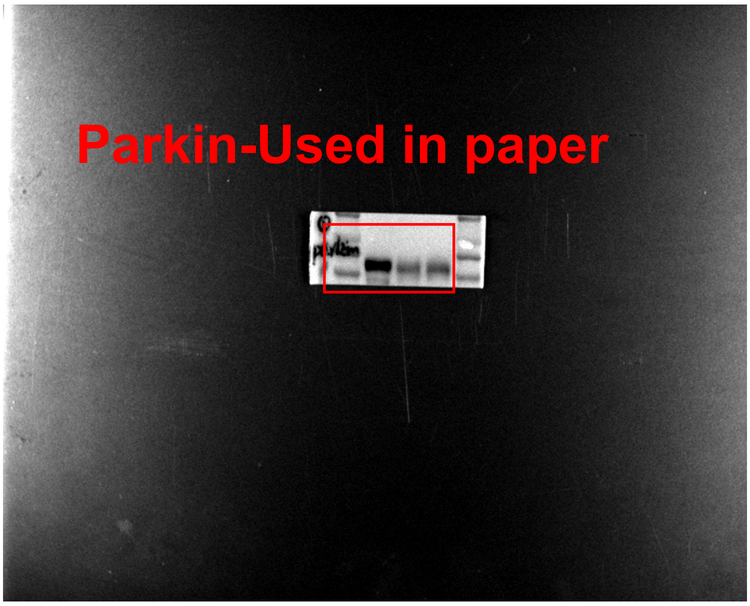


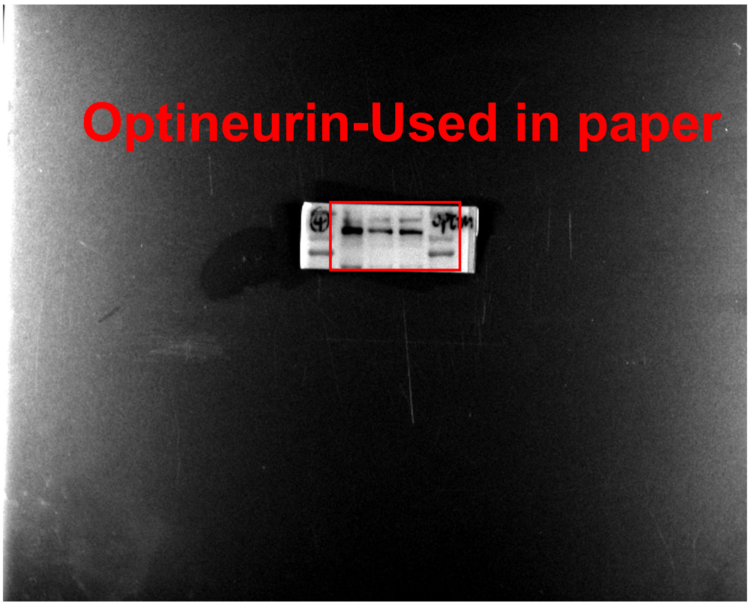


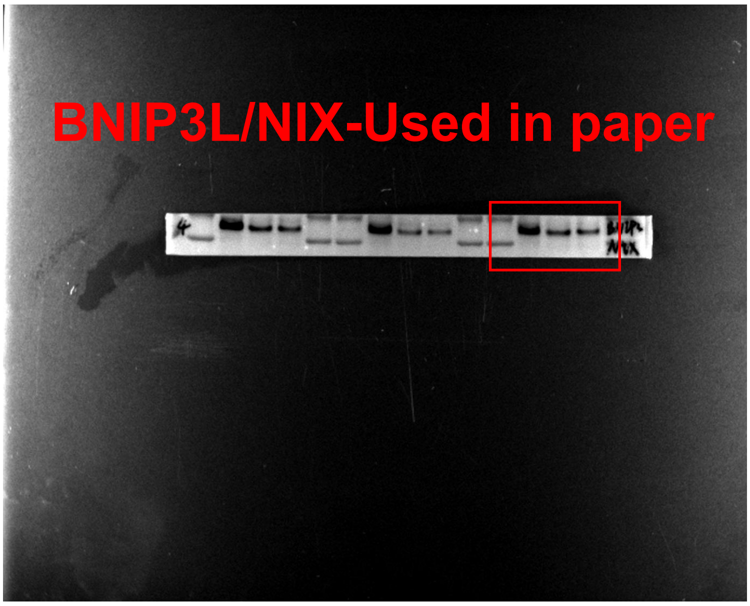

# Figure 4

G

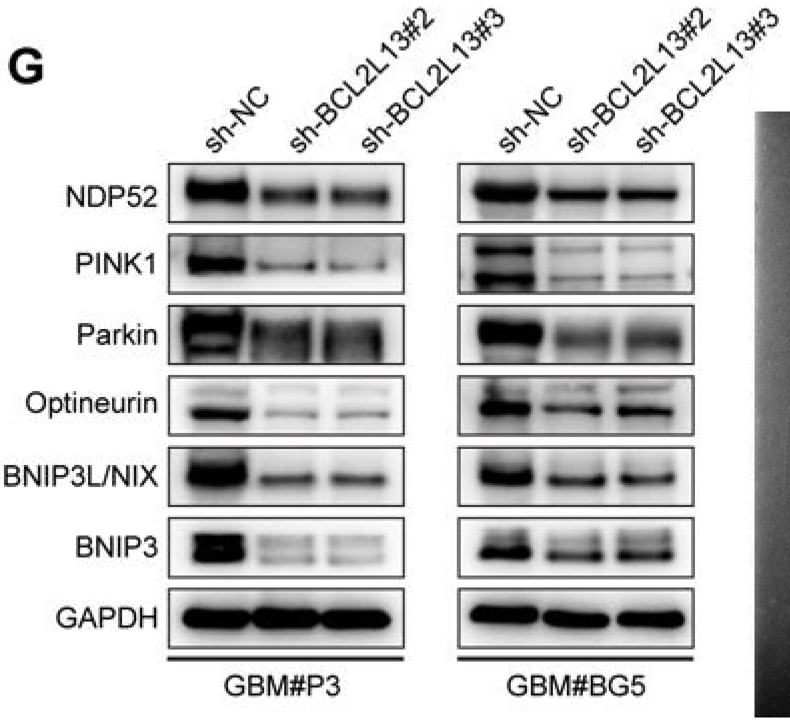


GBM#BG5

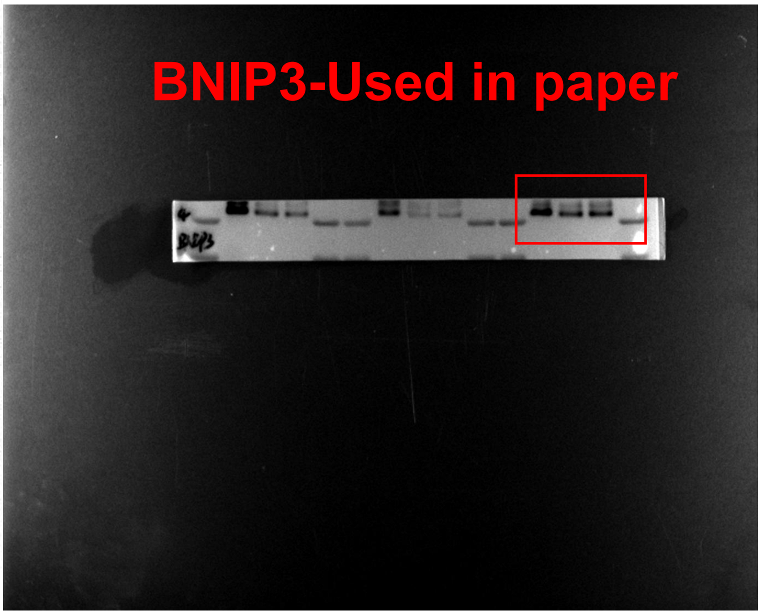


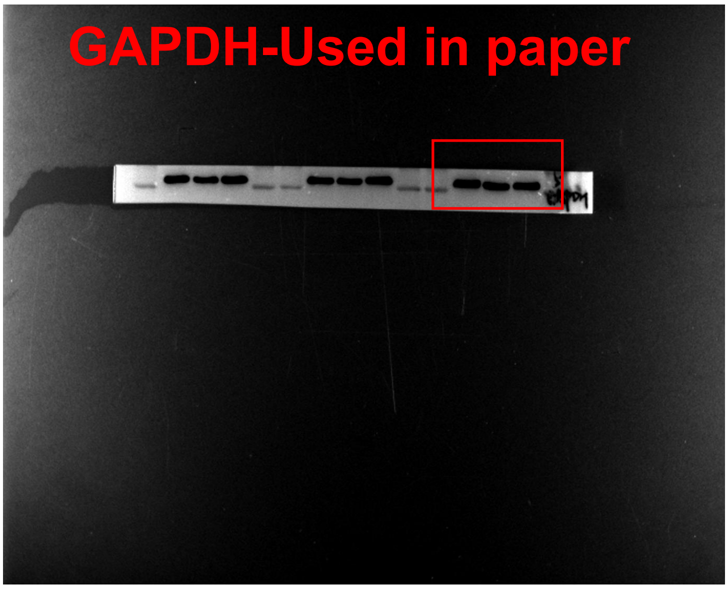

# Figure 5

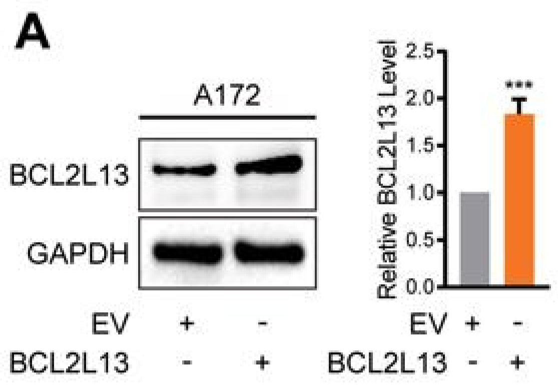
A
A172
BCL2L13
GAPDH
EV + -
BCL2L13 - +
Relative BCL2L13 Level
2.5
2.0
1.5
1.0
0.5
0.0
***
EV + -
BCL2L13 - +

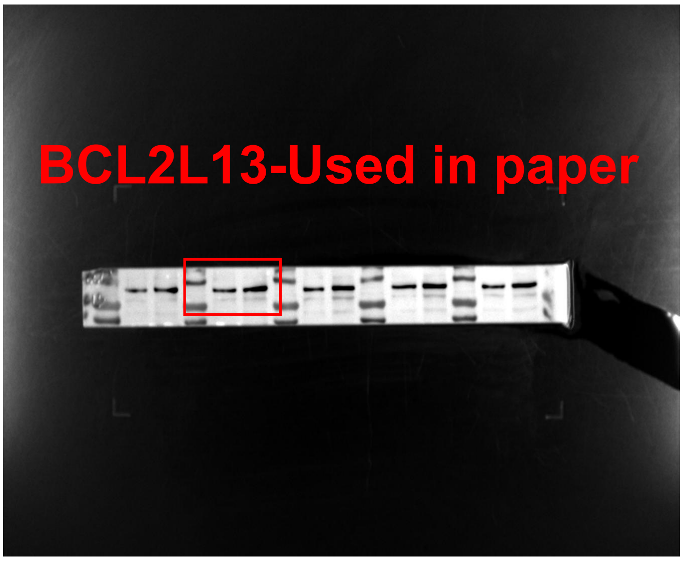
BCL2L13-Used in paper

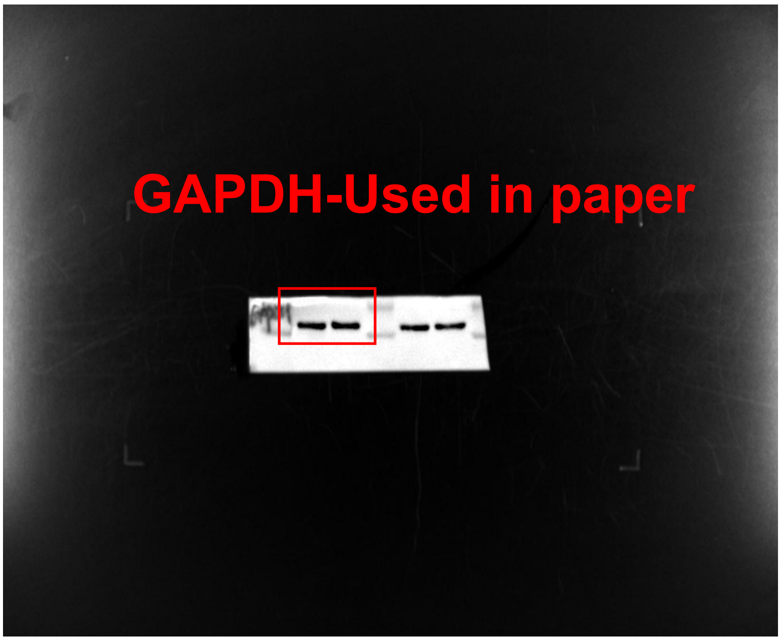
GAPDH-Used in paper

# Figure 5

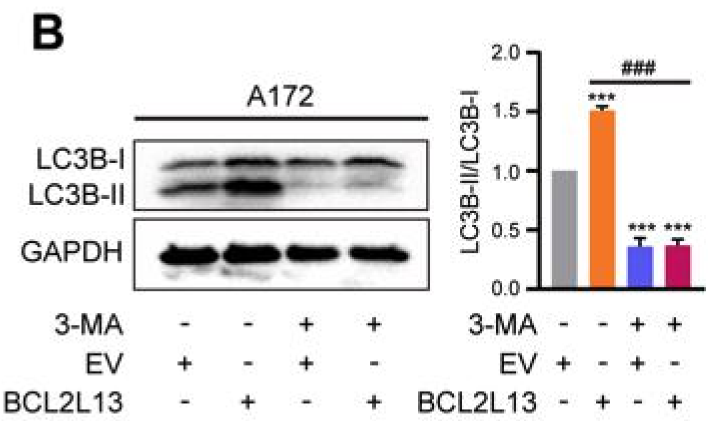
B
A172
LC3B-I
LC3B-II
GAPDH
3-MA - - + +
EV + - + -
BCL2L13 - + - +
LC3B-II/LC3B-I
2.0
1.5
1.0
0.5
0.0
###
***
*** ***
3-MA - - + +
EV + - + -
BCL2L13 - + - +

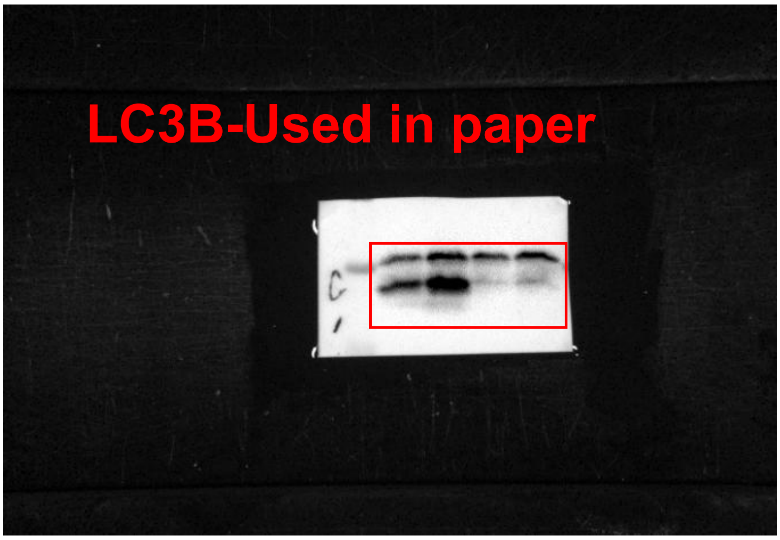
LC3B-Used in paper

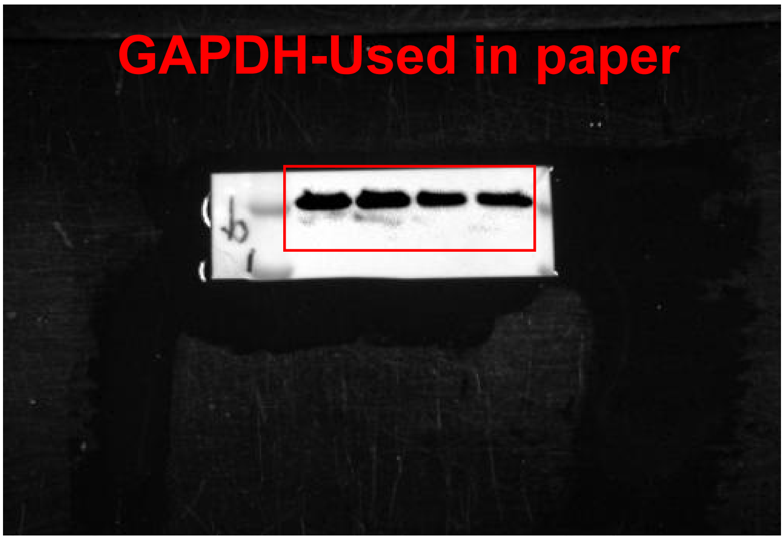
GAPDH-Used in paper

# Figure 5

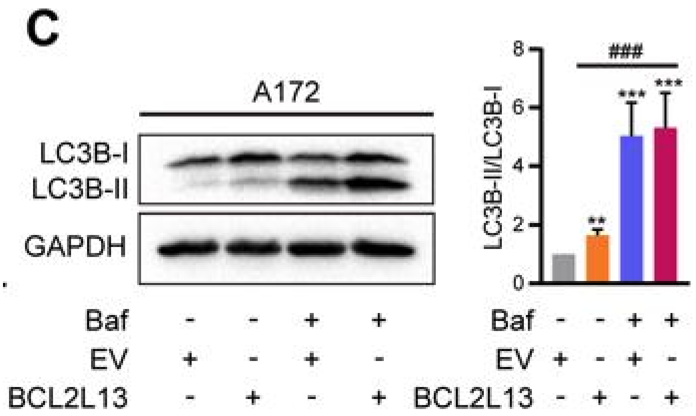
C
A172
LC3B-I
LC3B-II
GAPDH
LC3B-II/LC3B-I
###
***
**
Baf - - + +
EV + - + -
BCL2L13 - + - +

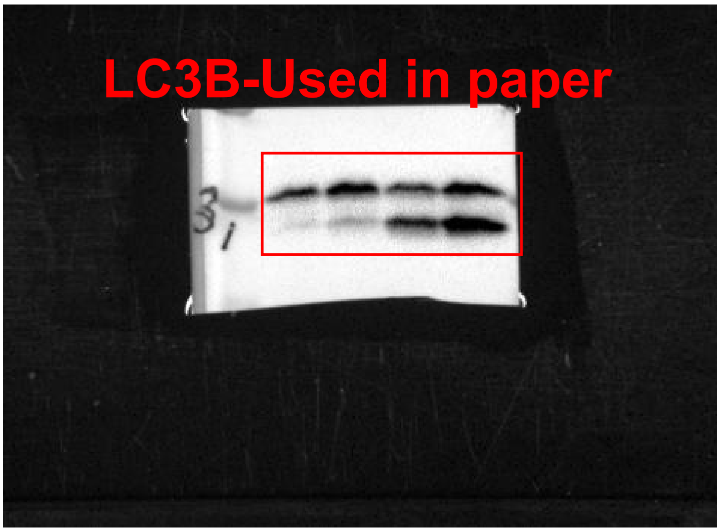
LC3B-Used in paper

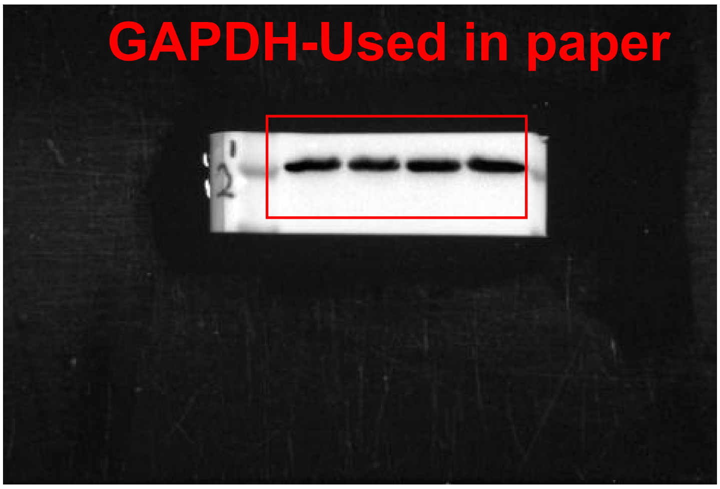
GAPDH-Used in paper

# Figure 6

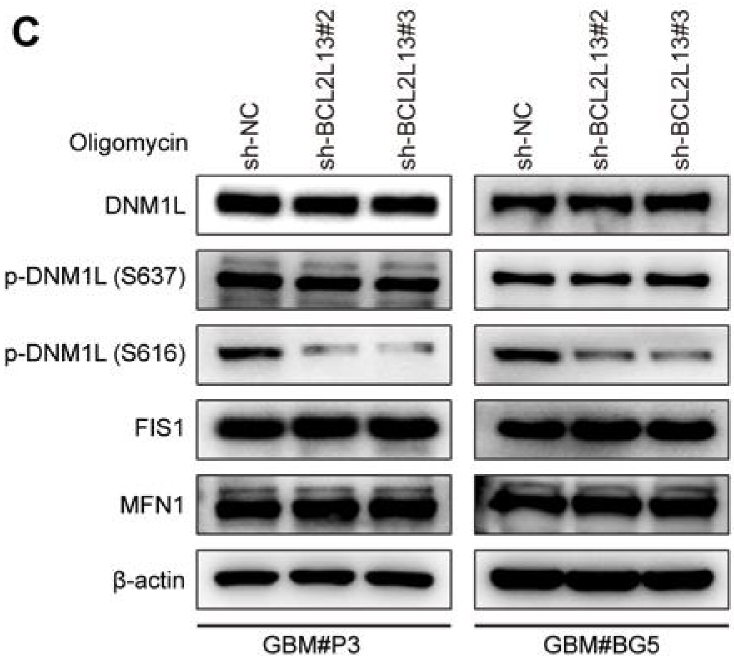


GBM#P3

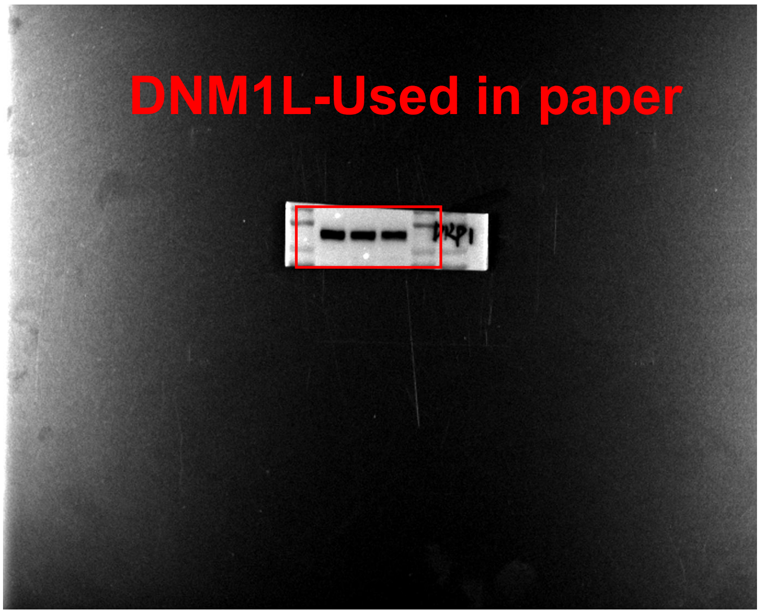


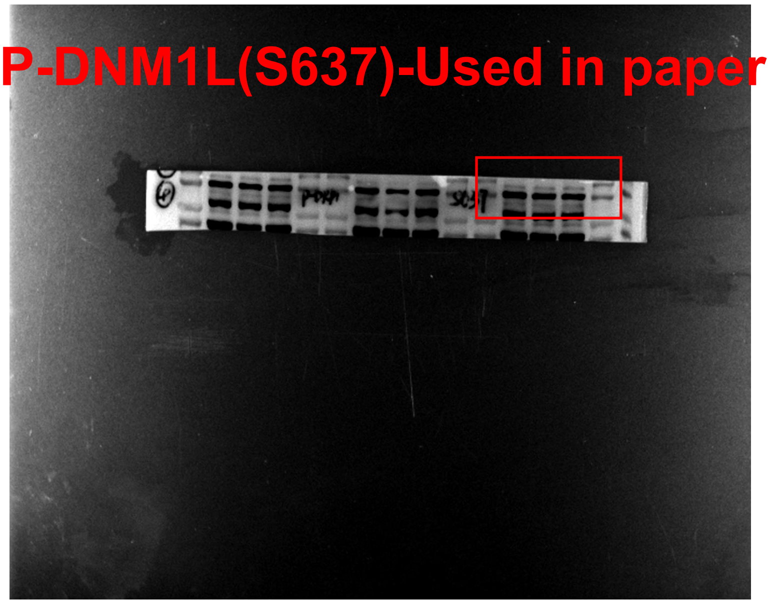


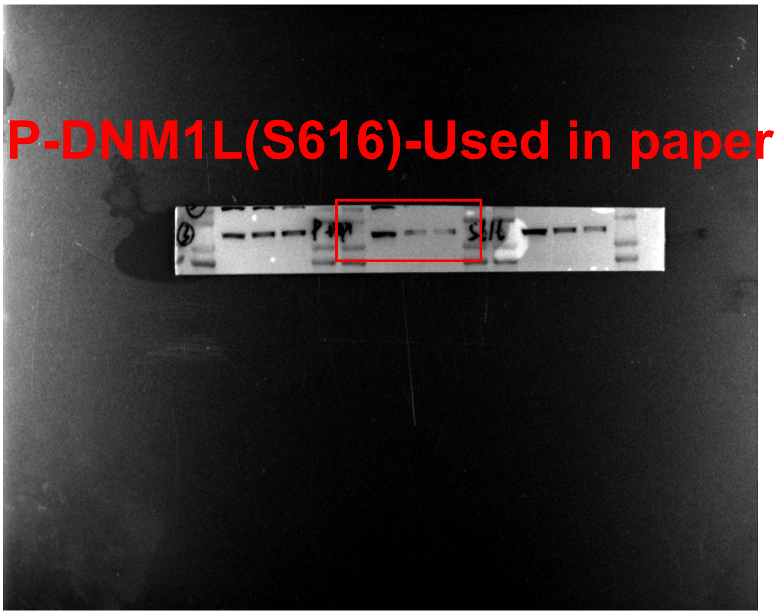


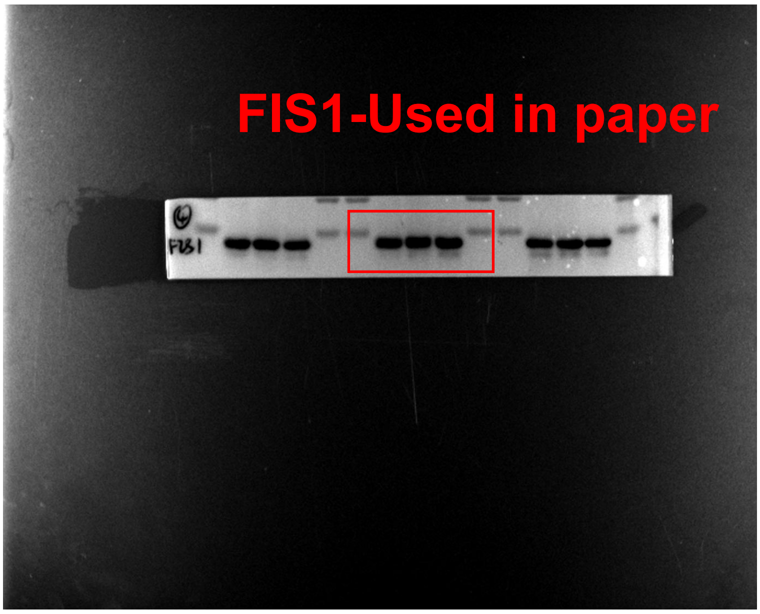


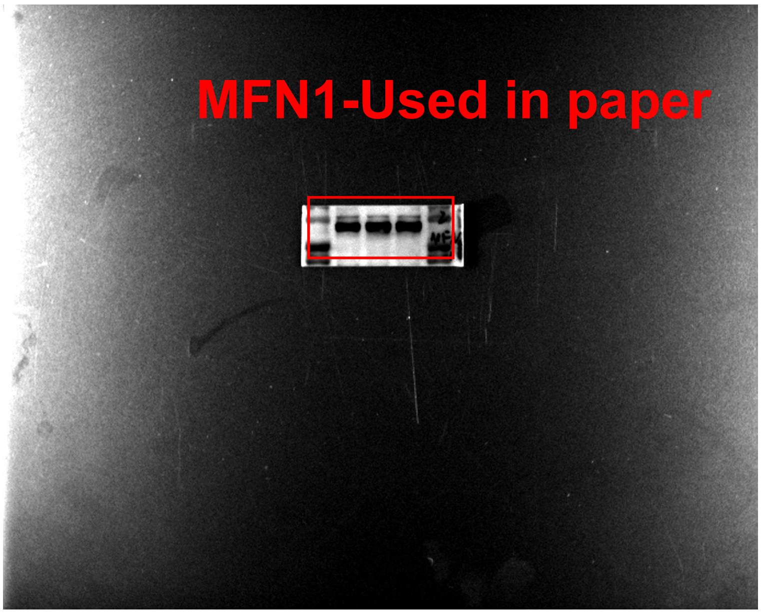

# Figure 6

GBM#P3

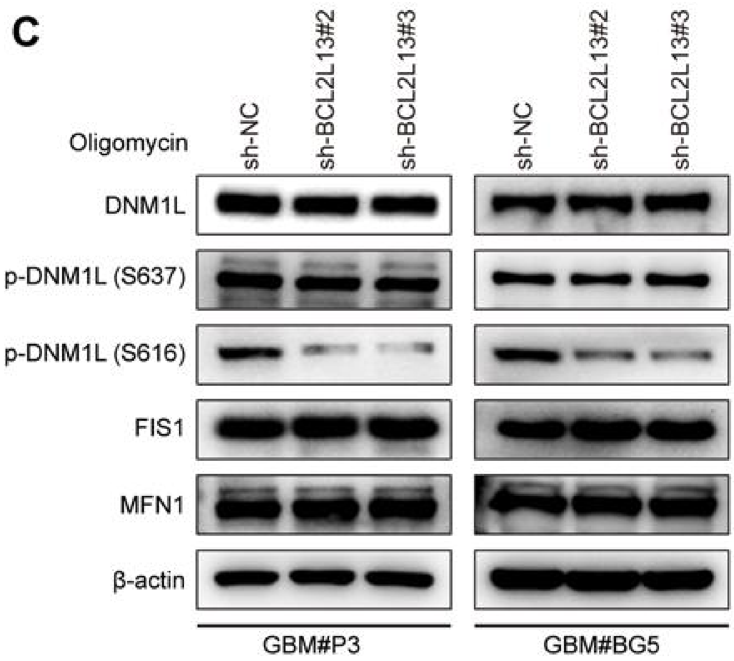


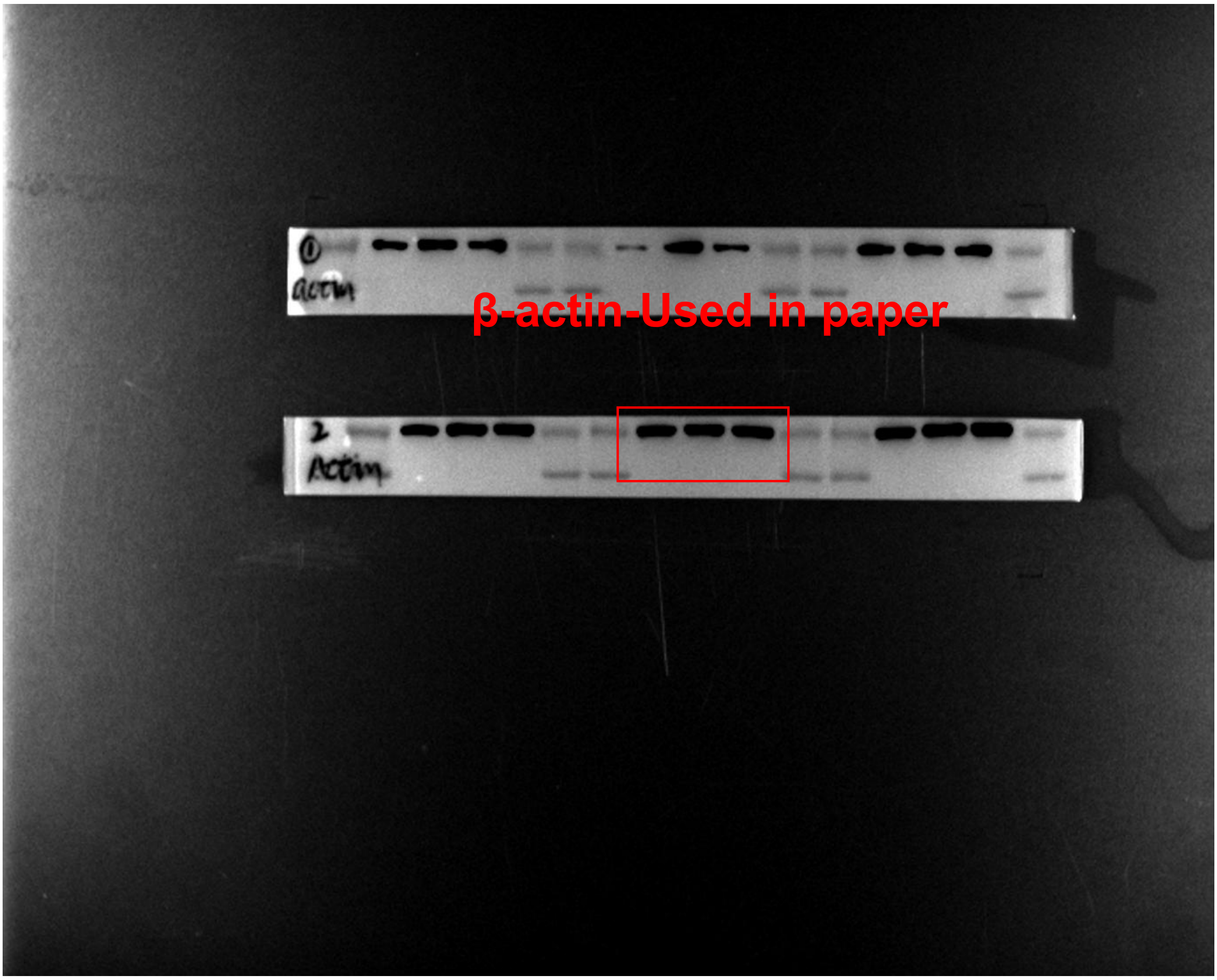

# Figure 6

C

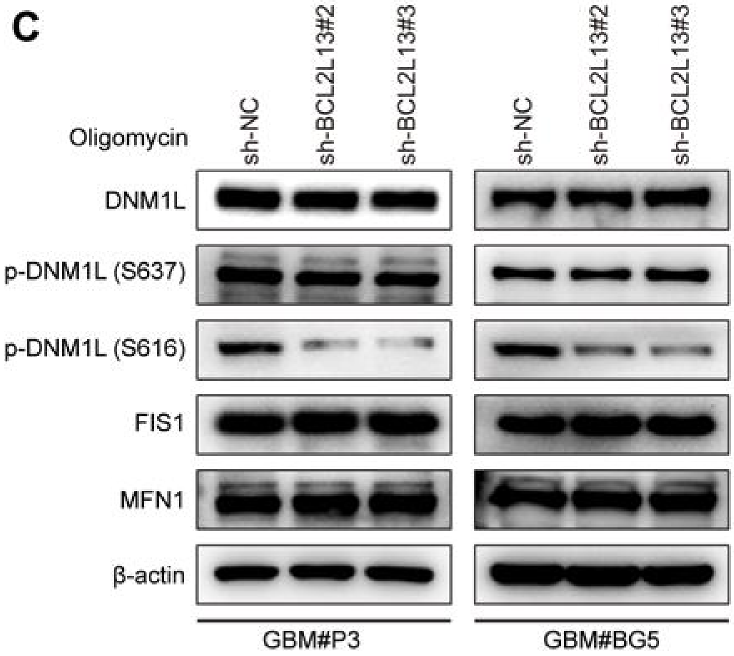


GBM#BG5

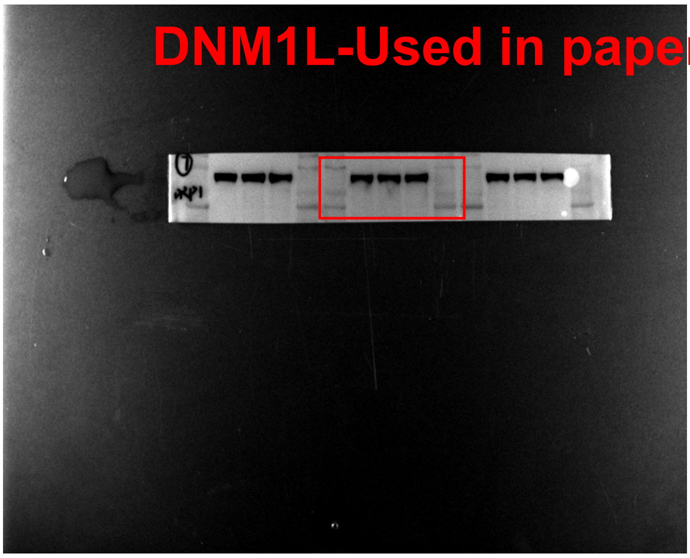


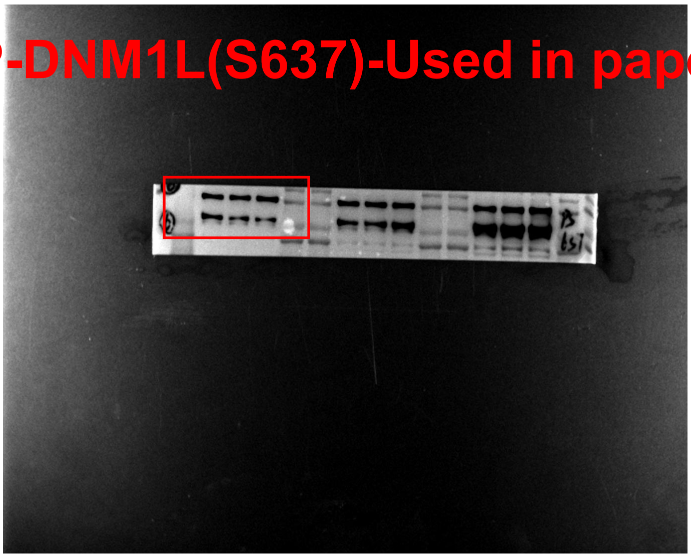


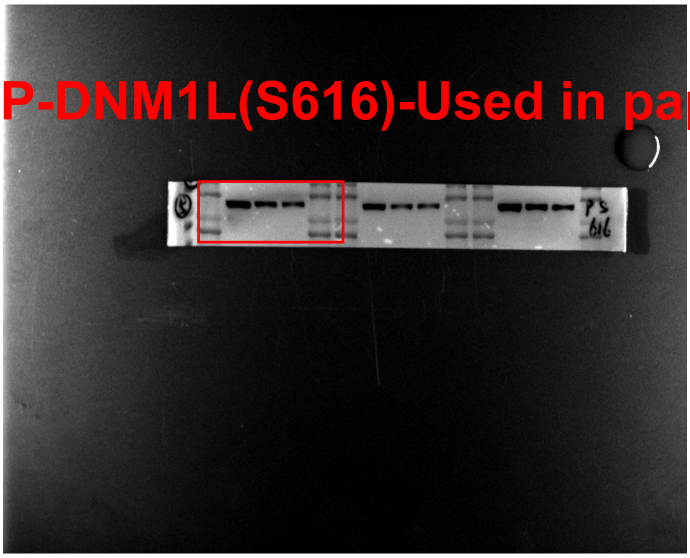


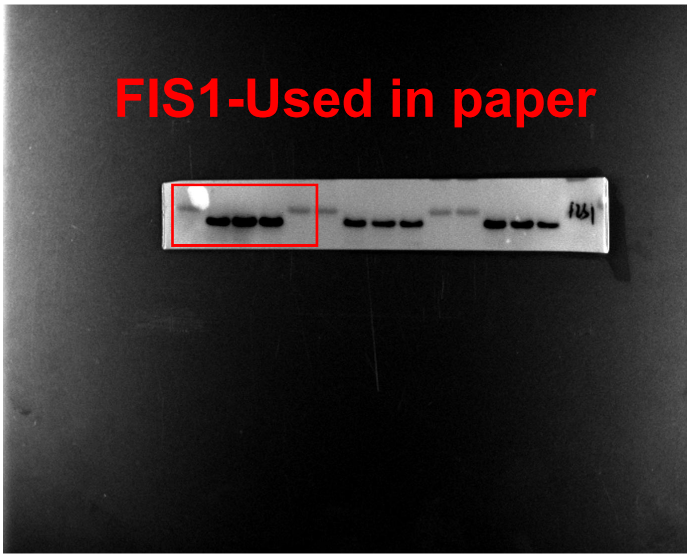


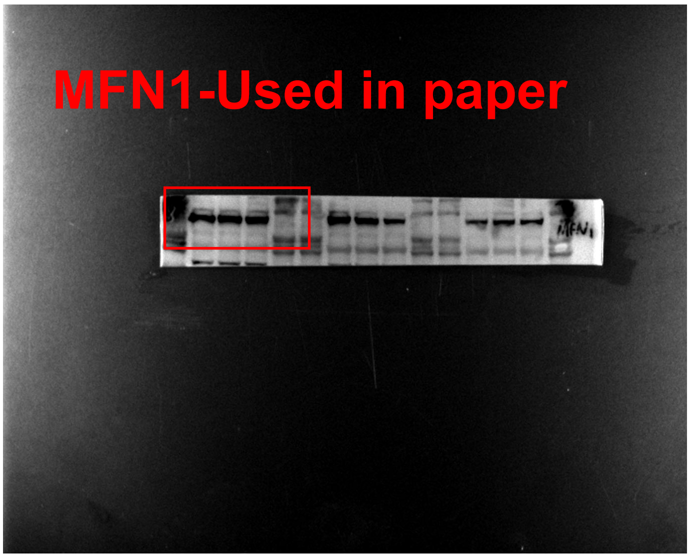

# Figure 6

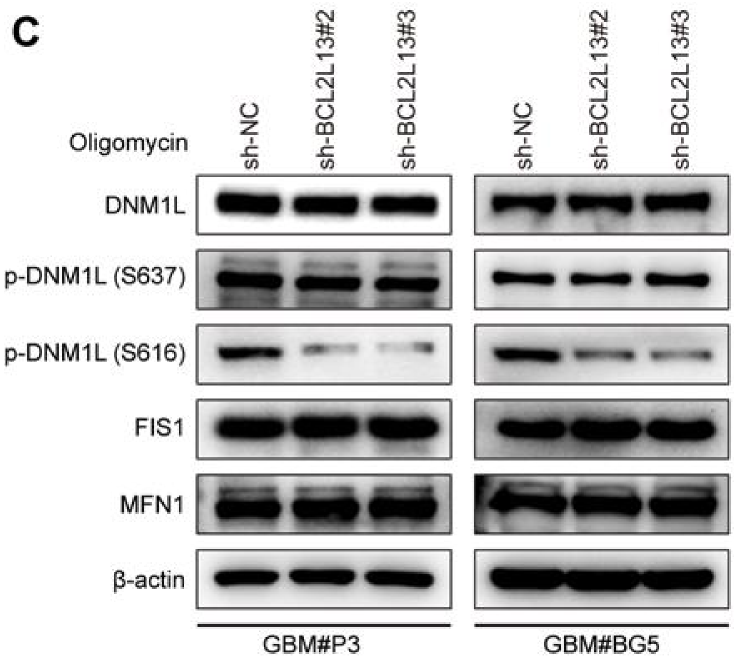


GBM#BG5

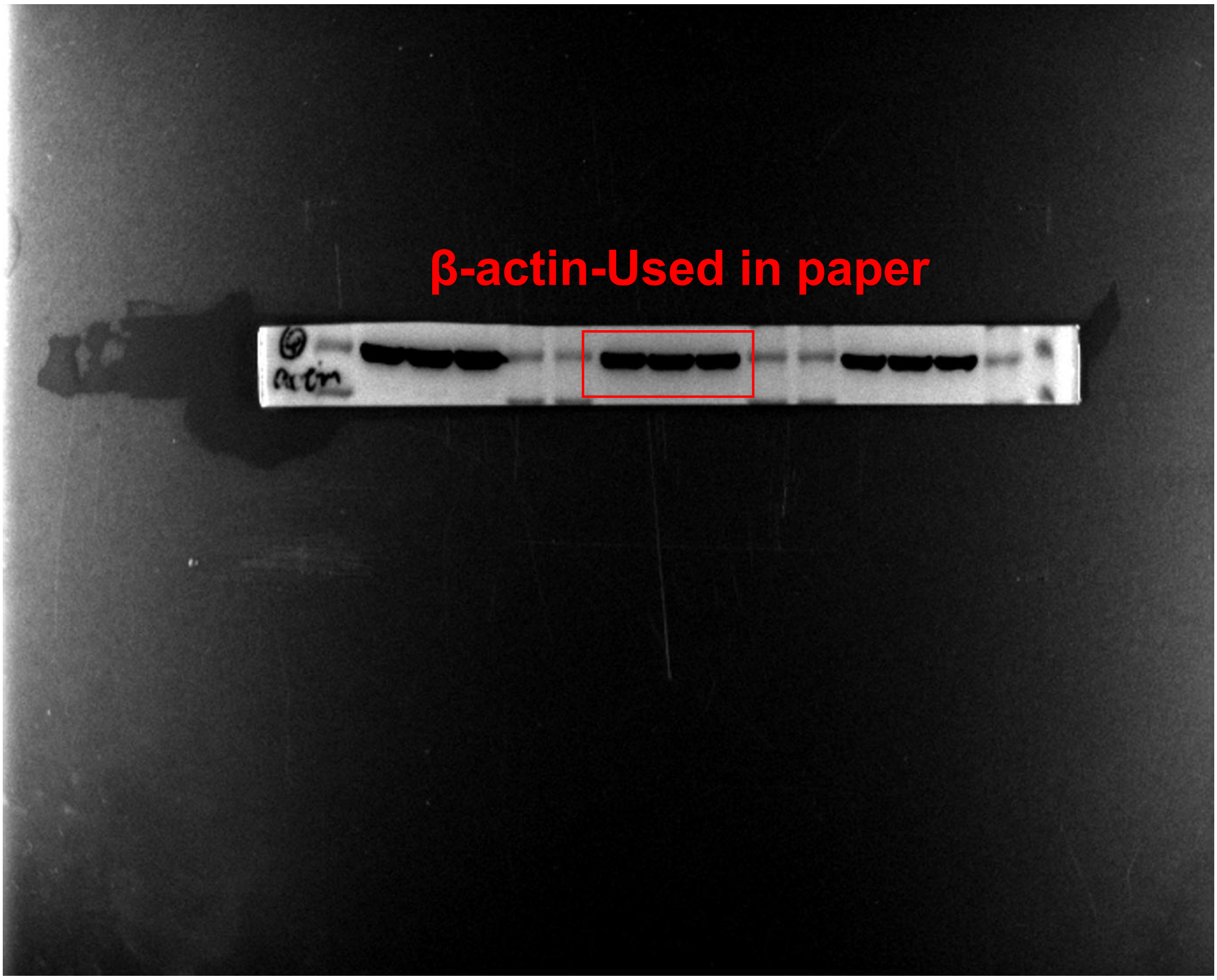

# Figure 7

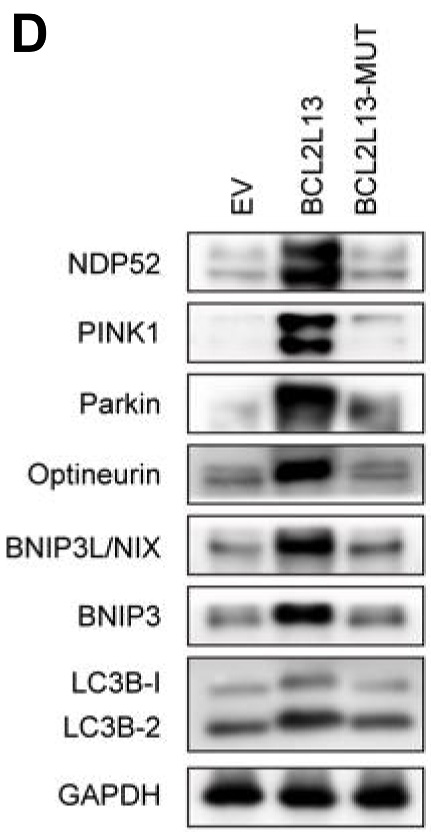
D
EV
BCL2L13
BCL2L13-MUT
NDP52
PINK1
Parkin
Optineurin
BNIP3L/NIX
BNIP3
LC3B-I
LC3B-2
GAPDH

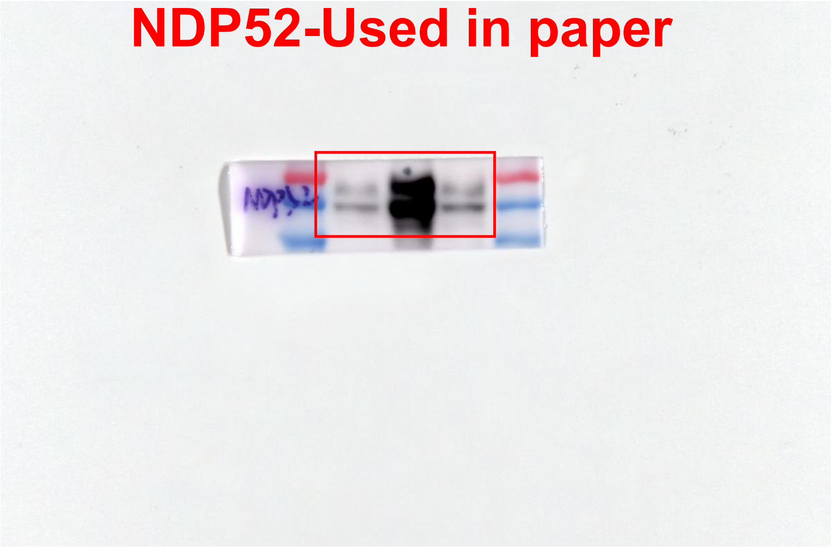
NDP52-Used in paper

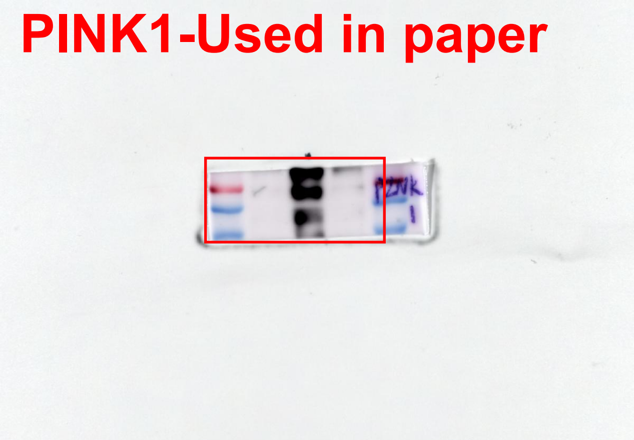
PINK1-Used in paper

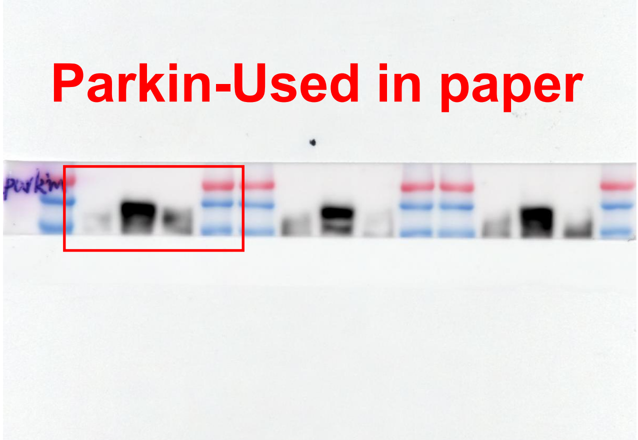
Parkin-Used in paper

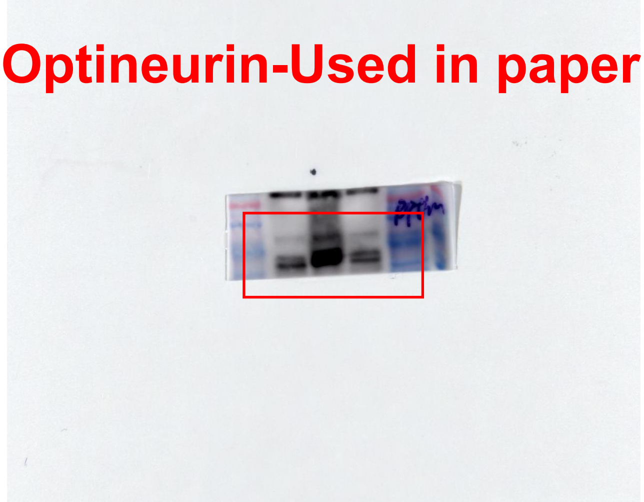
Optineurin-Used in paper

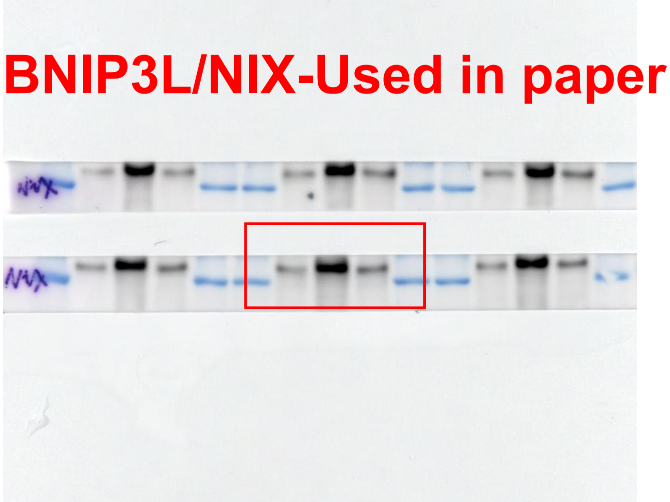
BNIP3L/NIX-Used in paper

# Figure 7

**D**

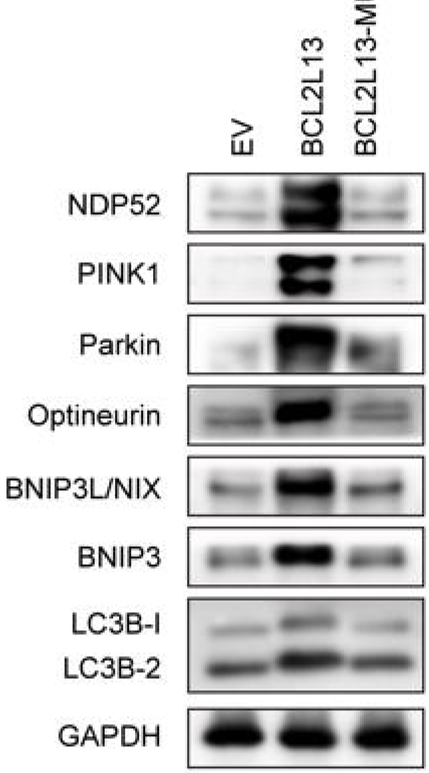
EV
BCL2L13
BCL2L13-MUT
NDP52
PINK1
Parkin
Optineurin
BNIP3L/NIX
BNIP3
LC3B-I
LC3B-2
GAPDH

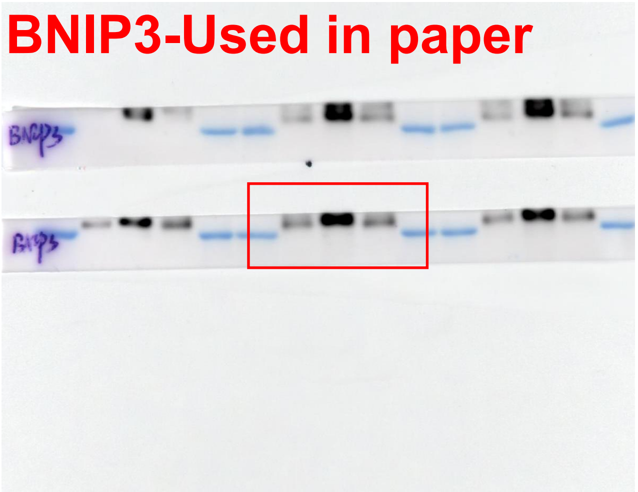
BNIP3-Used in paper

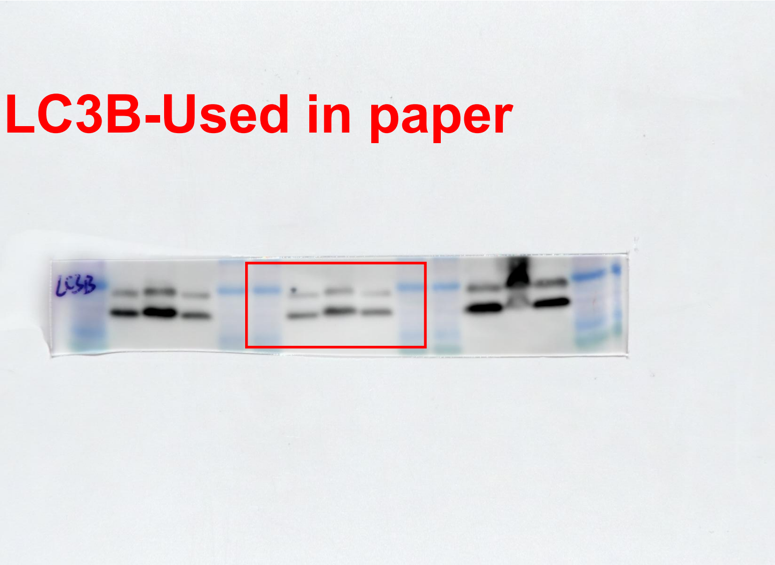
LC3B-Used in paper

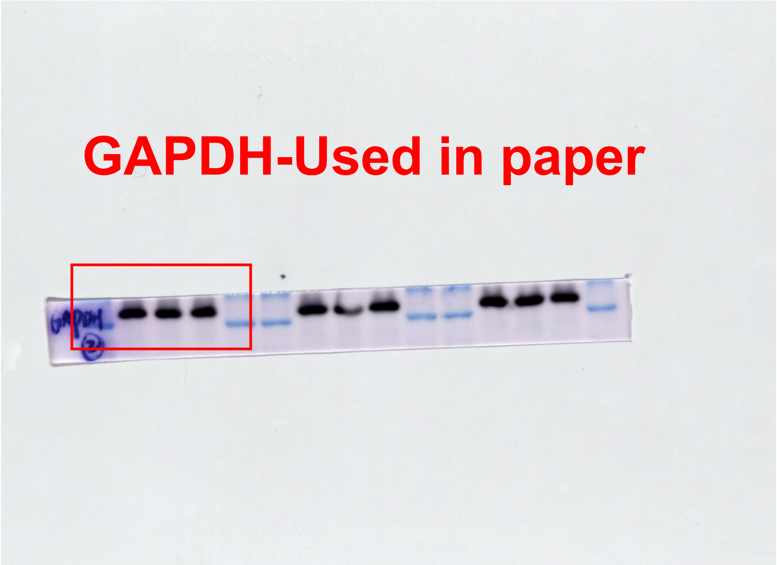
GAPDH-Used in paper

# Figure 7

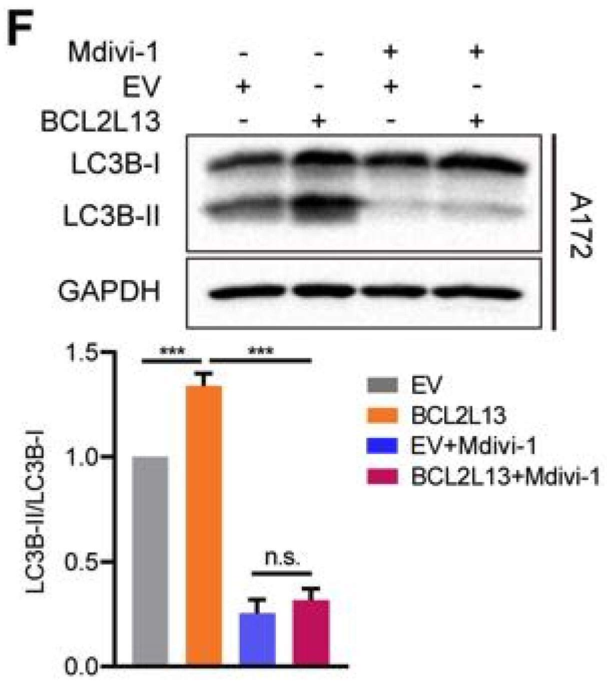
F
Mdivi-1
EV
BCL2L13
LC3B-I
LC3B-II
GAPDH
A172
LC3B-II/LC3B-I
1.5
1.0
0.5
0.0
***
***
n.s.
EV
BCL2L13
EV+Mdivi-1
BCL2L13+Mdivi-1

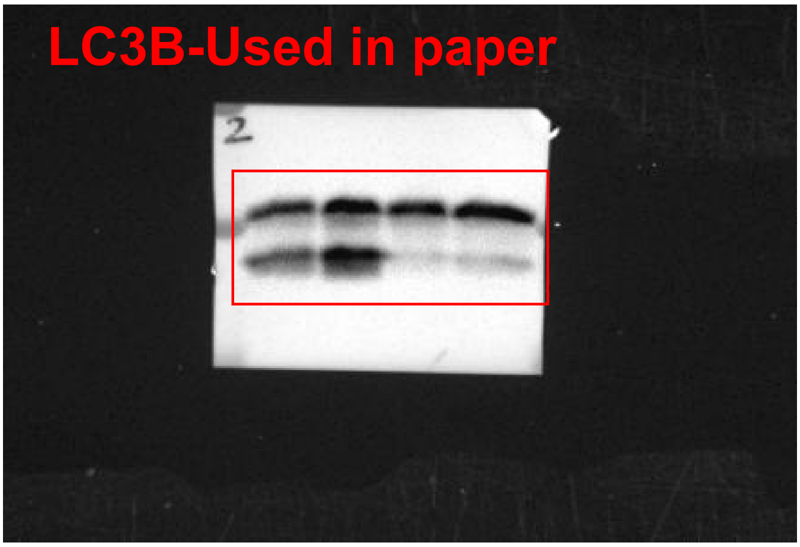
LC3B-Used in paper

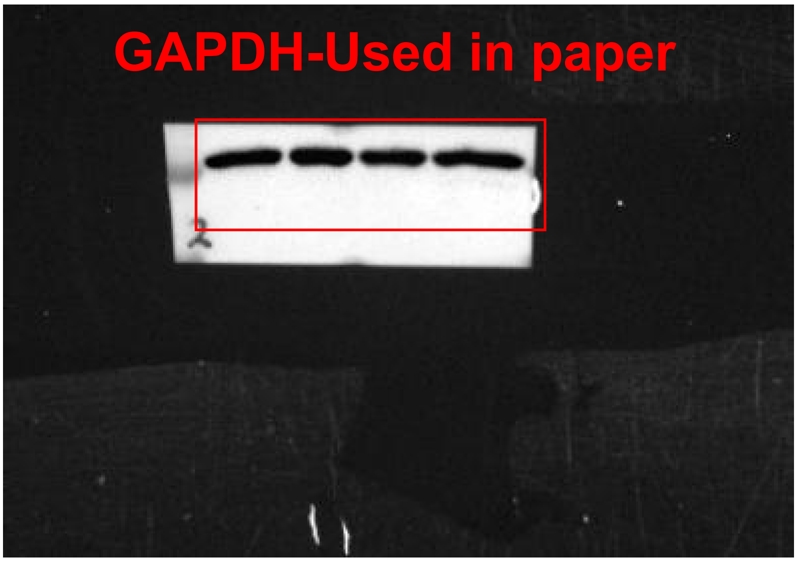
GAPDH-Used in paper
